# Highly stable Cas9 promotes HBV genome destruction by antagonizing HSC70-mediated degradation

**DOI:** 10.1080/22221751.2025.2556728

**Published:** 2025-09-04

**Authors:** Zhongqing Li, Yarong Song, Hongxin Huang, Ran Chen, Mingchen Liu, Xingwen Yang, Ziheng Luo, Benjamin M. Liu, Jie Wang

**Affiliations:** a Department of Microbiology & Infectious Disease Center, School of Basic Medical Sciences, Peking University Health Science Center, Beijing, People’s Republic of China; b NHC Key Laboratory of Medical Immunology, Peking University, Beijing, People’s Republic of China; c Department of Pathogen Biology and Biosecurity, Zhongshan School of Medicine, Sun Yat-sen University, Guangzhou, People’s Republic of China; d Division of Pathology and Laboratory Medicine, Children’s National Hospital, Washington, DC, USA; e Department of Pediatrics, School of Medicine and Health Sciences, The George Washington University, Washington, DC, USA; f Department of Microbiology, Immunology and Tropical Medicine, School of Medicine and Health Sciences, The George Washington University, Washington, DC, USA; g Children’s National Research Institute, Washington, DC, USA; h The District of Columbia Center for AIDS Research, Washington, DC, USA

**Keywords:** Highly stable Cas9, chaperone-mediated autophagy, heat shock cognate protein 70, hepatitis B virus

## Abstract

As a naturally existing adaptive immune system of prokaryotes against phages and foreign genetic materials, the CRISPR/Cas9 system has been widely used to combat various viral infections. However, its ability to destroy the constantly replicating viral genome and subsequently clear viral infections still needs further improvement. This study found that Cas9 protein was mainly degraded through the chaperone-mediated autophagy (CMA)-lysosome pathway in human cells, which was mediated by the binding between heat shock cognate protein 70 (HSC70) and Cas9 protein. HRS could stabilize Cas9 protein by competing with HSC70 to bind to Cas9 and subsequently inhibiting its degradation *via* the CMA-lysosome pathway. The stability of Cas9 protein with mutant KFERQ-like motifs located at aa 670–674 and aa 894–898 was significantly increased by antagonizing the HSC70-mediated CMA degradation, thus this Cas9 mutant was referred to as a highly stable Cas9 (HSCas9). The enhanced ability of HSCas9 to destroy the constantly replicating hepatitis B virus (HBV) genome promoted the CRISPR/Cas9 system to clear HBV infection without exhibiting cytotoxicity or increasing off-target effects. In summary, this study uncovers the degradation mechanism of Cas9 protein in human cells and provides a strategy to enhance the ability of the CRISPR/Cas9 system to clear HBV infection.

**Abbreviations:** ALP: autophagy-lysosome pathways; AR7: 7-Chloro-3-(4-methylphenyl)-2H-1,4-benzoxazine; cccDNA: covalently closed circular DNA; CMA: chaperone-mediated autophagy; CRISPR/Cas9: clustered regularly interspaced short palindromic repeats (CRISPR)/CRISPR-associated nuclease 9 (Cas9); gRNA: single-guide RNA; HBV: hepatitis B virus; HRS: hepatocyte growth factor-regulated tyrosine kinase substrate; HSC70: heat shock cognate protein 70; HSCas9: highly stable Cas9; rcDNA: relaxed circular DNA; SNP: single nucleotide polymorphism; UPS: ubiquitin-proteasome system

## Introduction

Clustered regularly interspaced short palindromic repeats (CRISPR)-/CRISPR-associated nuclease (Cas) system is a widespread prokaryotic defence system against invading phages and foreign genetic materials [1,2]. As one of the type II CRISPR/Cas systems, the CRISPR/Cas9 system, consisting of DNA endonuclease Cas9 and a single-guide RNA (gRNA) fused with CRISPR RNA (crRNA) and trans-activating crRNA, has been widely used in gene editing for almost all species [3,4]. As a naturally-existing adaptive immune system of prokaryotes, the CRISPR/Cas9 system has also been widely used to treat various viral infections in humans, including human immunodeficiency virus [5,6], herpes simplex virus [7], Epstein–Barr virus [8], cytomegalovirus [9], human papillomavirus [10], and hepatitis B virus (HBV) [11–19]. For HBV, we and other groups have found that the CRISPR/Cas9 system has a high efficiency in destroying the HBV genome [11–13], especially when using Cas9/gRNA ribonucleoprotein (RNP) complex *in vitro* [14–16]. However, its efficiency still needs to be further improved to clear HBV infection, especially when applied *in vivo* [17–19].

To reduce safety issues caused by off-target effects, the current priority for engineering the Cas9 protein is to shorten its half-life [20,21], which is indeed necessary for editing the non-replicating genome. However, the viral genome is constantly replicating in cells. For HBV, the complete HBV particles contain a partially double-stranded relaxed circular DNA (rcDNA) with a length of approximately 3.2 kb. After HBV infection, rcDNA enters the nuclei of hepatocytes and converts into covalently closed circular DNA (cccDNA), a template for producing progeny rcDNA. In turn, the newly synthesized progeny rcDNA can also re-enter the nuclei of hepatocytes to replenish the cccDNA pool [22]. To eradicate HBV infection, a more stable and efficient CRISPR/Cas9 system is needed to destroy the continuously replenished cccDNA. Although various chemical modifications can improve the stability of gRNA, a more stable and efficient Cas9 protein is still needed to clear viral infection. Previously, people were focusing on stabilizing the Cas9 protein. As an extracellular stable Cas9 protein, a thermostable GeoCas9 with an increased lifetime in human plasma improves the delivery of RNP *in vivo* [23]. As an intracellular stable Cas9 protein, Cas9-ubiquitin-associated domain (UBA) fusion protein can enhance the gene editing efficiency of the CRISPR/Cas9 system in plants by increasing the stability of Cas9 protein [24]. However, the gene editing ability of Cas9-UBA fusion protein in human cells may differ from that in plant cells, and the safety of Cas9-UBA fusion protein needs further investigation, as the fused UBA domain can lead to the polyubiquitin chain-induced phase separation, which may alter the function of the fusion protein [25]. Therefore, there is a greater need for a highly stable Cas9 (HSCas9) protein without fusion of additional domains.

Up to now, the degradation mechanism of Cas9 protein in human cells has not been fully elucidated. To develop an HSCas9 protein without fusion of additional domains, uncovering the degradation mechanism of the Cas9 protein in human cells is particularly important. In general, the degradation of intracellular proteins is mainly through the ubiquitin-proteasome system (UPS) and autophagy-lysosome pathways (ALP). In this study, we found that Cas9 protein was mainly degraded through ALP in human cells. Autophagy is a highly regulated cellular process that maintains cellular homeostasis through selective degradation of cytosolic proteins in the lysosome, which mainly consists of macroautophagy, microautophagy, and chaperone-mediated autophagy (CMA) [26]. In CMA, heat shock cognate protein 70 (HSC70) brings proteins with KFERQ-like motifs into lysosomes through a translocation complex composed of LAMP2A protein on the lysosomal membrane [27]. A KFERQ-like motif needs to meet (1) One or two of the following basic amino acids: K and R; (2) One of the following acidic amino acids: D and E; (3) One or two of the following hydrophobic amino acids: I, L, V, and F; (4) One hydrophilic amino acid Q on either side of the pentapeptide [27,28].

In this study, we found that HSC70-mediated CMA can promote the degradation of Cas9 protein in human cells. Based on this, we developed an HSCas9 protein that can antagonize HSC70-mediated CMA degradation, thereby enhancing its destructive activity against the continuously replicating HBV genome *via* its enhanced stability. Therefore, stabilizing the Cas9 protein can promote the CRISPR/Cas9 system to clear HBV infection, making it more clinically valuable in antiviral therapy.

## Materials and methods

### Cell culture, transfection, and treatment

HuH7, HepG2, and HeLa cells were maintained in a Dulbecco’s modified Eagle medium (DMEM) (Corning, New York, USA) supplemented with 10% fetal bovine serum (FBS) (PAN, Adenbach, Bavaria, Germany) and 1% penicillin/streptomycin (PS), in a 5% CO_2_ incubator at 37℃. The stable HBV-expressing cell line HepAD38 was kindly provided by Prof. Ningshao Xia at Xiamen University, and was cultured with DMEM containing 10% FBS, 400 μg/mL G418 (Amresco, Solon, Washington, USA), and 4 μg/mL Doxycycline (Merck, Kenilworth, New Jersey, USA). HepG2-NTCP cells were kindly provided by Prof. Kuanhui Xiang at Peking University and were cultured with DMEM containing 10% FBS and 400 μg/mL G418. *LAMP2A* gene knockout (KO) HEK293 T cells, *SQSTM1/p62* gene KO HEK293 T cells, *ATG3* gene KO HEK293 T cells, *ATG5* gene KO HEK293 T cells, and the corresponding control cells were constructed [29] and kindly provided by Prof. Hongxin Huang at Sun Yat-sen University, and were cultured with DMEM containing 10% FBS, 1% PS, and 1 μg/mL puromycin (Thermo Fisher Scientific, Waltham, Massachusetts, USA). HeLa-Cas9-Flag cells stably expressing Cas9 [30], HepG2-NTCP-Cas9 cells stably expressing Cas9 and HepG2-NTCP-HSCas9 cells expressing HSCas9 were constructed in our laboratory and were cultured with DMEM containing 10% FBS, 1% PS, and 1 μg/mL puromycin (Thermo Fisher Scientific, Waltham, Massachusetts, USA).

Lipofectamine 2000 (Invitrogen, Carlsbad, CA, USA) was used to transfect the plasmid, and Lipofectamine RNAiMAX (Thermo Fisher Scientific, Waltham, MA, USA) was used to transfect siRNA oligonucleotide duplex (RiBobio, Guangzhou, Guangdong, China), according to the manufacturer's protocol. MG132 (Selleck, Houston, TX, USA), HCQ (Selleck, Houston, TX, USA), BafA1 (MCE, New Jersey, USA), Cycloheximide (MCE, New Jersey, USA), AR7 (MCE, New Jersey, USA), and LY294002 (MCE, New Jersey, USA) were used to treat cells, respectively.

### Plasmids

The plasmid pBB4.5-HBV1.3 was previously constructed in our laboratory. The PCDH-HSC70-HA plasmid was constructed by amplifying the HSC70 coding sequence attached with an HA tag and inserting it into the PCDH plasmid digested with BamHI and XbaI. Based on the pSpCas9 (BB)-2A-EGFP (PX458) plasmid (Addgene, Cambridge, MA), the PX458-Cas9M1, PX458-Cas9M2, PX458-Cas9M3, PX458-Cas9M4, and PX458-HSCas9 plasmids were constructed using site-directed mutagenesis using the QuikChange site-directed mutagenesis kit (Stratagene, La Jolla, CA). Based on the lentiCRISPRv2 plasmid (Addgene, Cambridge, MA), the lenti-HSCas9 plasmid was constructed by site-directed mutagenesis. The primer sequences used for plasmid construction are shown in Supplementary Table S1.

### RT-qPCR

Reverse transcription was performed using the Transcriptor First Strand cDNA Synthesis Kit (Roche, Basel, Kanton Basel, Switzerland). The mRNA levels of *Cas9*, *LAMP2A*, and *ACTB* were detected by reverse transcription-quantitative polymerase chain reaction (RT-qPCR) (SYBR Green method) using an Applied Biosystems Step One Plus Real-Time PCR system (Thermo Fisher Scientific, Waltham, Massachusetts, USA). The primers used to detect the levels of gRNA and mRNA are listed in Supplementary Table S2.

### On-target cleavage efficiency analysis

The total DNA of cells was extracted using QIAamp DNA Mini Kit (Qiagen, Hilden, Germany), according to the manufacturer’s instructions. The on-target cleavage efficiency of the HBV genome was assessed by PCR using the primers outside of the two cleavage sites of gHBV1/Cas9 and gHBV2/Cas9, HBV-F (5′-GACTCGTGGTGGACTTCTCTCAA-3′), and HBV-R (5′-CTGACTACTAATTCCCTGGATGCT-3′). The PCR products were detected by 1.5% agarose gel electrophoresis and purified for sequencing by NucleoSpin Gel and PCR Clean-up Kit (Macherey-Nagel). The purified PCR products were sequenced by Sangon Biotech (Shanghai, China).

Further, the on-target cleavage efficiency of the HBV genome was assessed by the next-generation sequencing. Specifically, the HBV genome was amplified with the primers outside of the two cleavage sites of gHBV1/Cas9 and gHBV2/Cas9, HBV-F1 (5′-ATCCTGCCTTGATGCCTTTA-3′), and HBV-R1 (5′-ACAGAGCTGAGGCGGTGTCG-3′). The next-generation sequencing of PCR products was performed by Sangon Biotech (Shanghai, China), and 150 bp paired-end reads were generated. The raw reads were filtered by Trimmomatic (Version 0.39) to obtain clean reads. Clean reads were aligned to the HBV reference genome using a Burrows–Wheeler Aligner (BWA, Version 0.7.17). After alignment, SAMtools (Version 1.9) was used to convert the alignment result format, and GATK (Version 4.1.1.0) was used to call out all variants, including insertion or deletion (indel) variants. The on-target cleavage efficiency of the HBV genome was the number of indel variant reads at the cleavage site divided by the total number of reads at the cleavage site.

### HBV cccDNA detection

The nuclear DNA was extracted as the protocol previously reported [31]. Briefly, cells were lysed with TE buffer and 10% SDS. The cell lysates were added with 5 M NaCl and were precipitated for at least 16 h at 4°C, and then the supernatant was collected after centrifugation. Finally, the nuclear DNA was purified by phenol/chloroform and ethanol. The level of the complete HBV cccDNA was detected by qPCR using the primers between the two cleavage sites of gHBV1/Cas9 and gHBV2/Cas9, cccDNA-F: GGGGCGCACCTCTCTTTA, and cccDNA-R: AGGCACAGCTTGGAGGC.

### Detection of HBsAg and HBeAg

The levels of HBsAg and HBeAg were detected by chemiluminescence immunoassay kits (Autobio Diagnostics Co., Zhengzhou, China), according to the manufacturer's instructions.

### Co-immunoprecipitation (Co-IP) assay and Western blotting

Co-IP assay and Western blotting were performed as we previously described [32]. The protein levels were detected by the chemiluminescence imaging analysis system (Tanon, Shanghai, China) or the Odyssey infrared imaging system (LICOR, Lincoln, Nebraska, USA). The corresponding antibodies are shown in Supplementary Table S3.

### Immunofluorescence (IF) assay

Cells were cultured on the Petri dish for confocal (D35-20-1-N, Cellvis), and then fixed with 4% PFA and permeabilized with 0.1% TritonX-100 or Tween-20. Subsequently, the cells were incubated with the primary antibodies at 4°C overnight. After washing three times with 1 × PBS, the cells were incubated with the secondary antibodies for 1 h at room temperature, and then were observed under a STELLARIS 8 confocal laser scanning microscope (Leica, Frankfurt, Hesse-Darmstadt, Germany). The corresponding antibodies are shown in Supplementary Table S3.

### Cell counting kit-8 assay

A total of 1 × 10^4^ cells were seeded in 96-well plates with six duplicates, and then the cell viability was detected using a Cell Counting Kit-8 (CCK-8) (Dojindo, Japan). The absorbance was measured at the wavelength of 450 nm by a SpectraMax Absorbance Reader (Molecular Devices, California, USA).

### Off-target analysis

The off-target efficiency of Cas9 was assessed by the next-generation sequencing after PCR. Specifically, the off-target sites of gHBV1 in the human genome (GRCh38/hg38) were predicted by the Cas-OFFinder tool [33]. The top three predicted off-target sites were selected and listed in Supplementary Table S4. The primers used for PCR amplification are listed in Supplementary Table S5. The PCR products were sequenced by Sangon Biotech Co., Ltd. (Shanghai, China). The paired-end sequencing of the libraries was performed on the Illumina sequencing platform (NovaSeq6000, USA). Three steps filtered the raw reads: (1) Removing adaptor sequence by the Cutadapt software; (2) Removing the reads with a mean quality score <20 by PRINSEQ-lite software; (3) Removing chimaeras’ sequence by Usearch software. The analyses and visualization of the off-target effects were performed by CRISPResso2. The off-target efficiency was the number of reads containing an indel variant divided by the total number of reads.

The potential off-target sites of gHBV1 and gHBV2 in the mouse genome (mm39) were also predicted by the Cas-OFFinder tool. Based on the whole-genome sequencing data, single-nucleotide polymorphism (SNP), and indel variants within 20 bp upstream and downstream of all potential off-target sites were identified and analysed.

### Southern blotting

Southern blotting was performed using the DIG High Prime DNA Labelling and Detection Starter Kit II (Roche, Basel, Kanton Basel, Switzerland). Briefly, HBV cccDNA was separated in a 1.2% agarose gel. After denaturalization and neutralization, DNA was transferred from the gel onto a Nylon membrane (Roche, Basel, Kanton Basel, Switzerland). Then the membrane was cross-linked with UV energy dosage at 120 mJ/cm^2^ and then hybridized overnight at 42°C with a DIG-labelled HBV probe. Subsequently, the membrane was blocked and incubated with anti-digoxigenin-AP Fab fragments, followed by exposure via a ChemiScope6100 imaging device (Clinx, Shanghai, China).

### HBV infection

HBV was collected from the supernatants of HepAD38 cells, followed by concentration and HBV DNA quantification. HepG2-NTCP-Cas9 and HepG2-NTCP-HSCas9 cells were seeded in a 6-well plate and were transfected with pU6-t-g1-t-g2-t plasmid or vector control. At 24 h post-transfection, the cells were inoculated with 500 HBV genome equivalents per cell (500 geq/cell) in the presence of 4% PEG8000 and 2% DMSO for 24 h. After HBV infection, the cells were washed with PBS 5 times, and the fresh medium with DMEM containing 2% DMSO and 2% FBS was added. Five days later, the cells and supernatants were collected for detection.

### Hydrodynamic injection mouse model

Hydrodynamic injection experiments were carried out as we previously reported [34]. Twenty-four male C57BL/6 mice (5 weeks old) were randomly divided into NC, Cas9, and HSCas9 groups, and there were 8 mice in each group. The mice in the NC group were injected with pBB4.5–1.3 × HBV, PX458, and pU6 plasmids. The mice in the Cas9 group were injected with pBB4.5–1.3 × HBV, PX458, and pU6-t-g1-t-g2-t plasmids. The mice in the HSCas9 group were injected with pBB4.5–1.3 × HBV, PX458-HSCas9, and pU6-t-g1-t-g2-t plasmids. Sera were collected at 5 and 7 days after hydrodynamic injection, and liver tissues were collected at 3 and 7 days after hydrodynamic injection, respectively.

Animal experiments were approved by the Ethics Committee of Peking University and were carried out according to the guidelines established by the Institutional Animal Care and Use Committee at Peking University Health Science Center.

### Whole-genome sequencing (WGS)

Genomic DNA of three mice per group was extracted using QIAamp DNA Mini Kit (Qiagen, Hilden, Germany), according to the manufacturer’s instructions. Library construction and sequencing were conducted by OE Biotech. (Shanghai, China). The raw reads were subjected to a quality check and then filtered by FASTQ (Version 0.19.5) to obtain clean reads. Clean reads were aligned to the mouse (mm39) reference genome using a Burrows–Wheeler Aligner (BWA, Version 0.7.12). After alignment, Picard (http://broadinstitute.github.io/picard/, Version 4.1.0.0) was employed to mark duplicate reads, and SAMtools (Version 1.4) was used to convert the alignment result format. GATK (Version 4.1.0.0) was used to call out all variants, including SNPs and indel variants. Further, the variants (SNPs and indels) shared by all samples were removed to obtain the confidence variants.

### Detection of alanine aminotransferase (ALT) and aspartate aminotransferase (AST)

The levels of ALT and AST in the sera of mice were detected by ELISA kits (Elabscience, Wuhan, China), according to the manufacturer's instructions.

### Statistical analysis

All data were presented as mean ± standard deviation (SD) of three independent experiments. Statistical analyses were performed by SPSS 24.0 software. Comparisons of the differences between groups were analysed by two-tailed Student’s *t*-tests, and a *P*-value less than 0.05 was considered statistically significant.

## Results

### Cas9 protein is primarily degraded through the HSC70-mediated CMA-lysosome pathway

**Figure 1.**
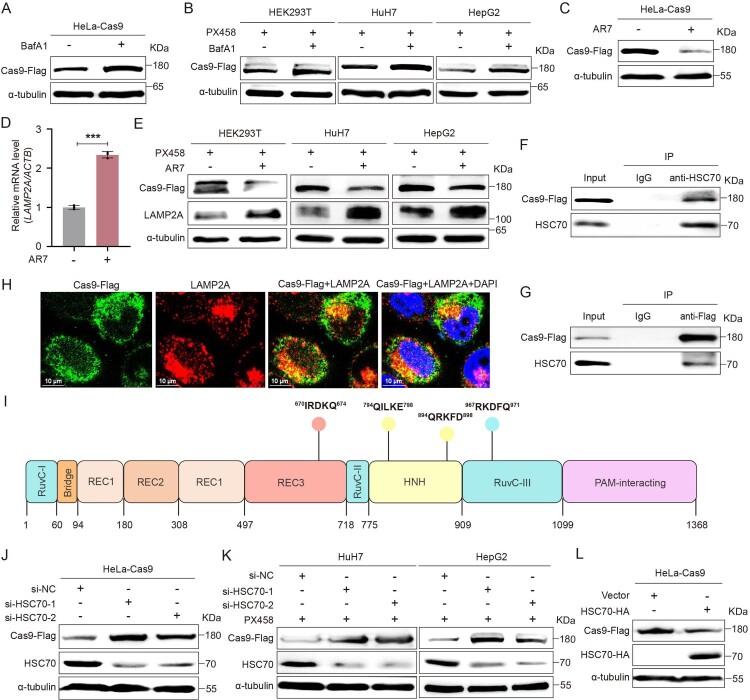
Cas9 protein was degraded through the HSC70-mediated CMA-lysosome pathway. (A) HeLa-Cas9 cells were treated with BafA1 (10 nM) for 12 h, and the level of Cas9 protein was detected by Western blotting. (B) HEK293 T, HuH7, and HepG2 cells were transfected with Cas9 expression plasmid PX458 and treated with BafA1 (10 nM) for 12 h after 48 h of transfection, and the level of Cas9 protein was detected by Western blotting. HeLa-Cas9 cells were treated with AR7 (20 µM) for 24 h. (C) The level of Cas9 protein was detected by Western blotting; (D) the level of *LAMP2A* mRNA was detected by RT-qPCR, and *ACTB* mRNA was used as the internal control for RT-qPCR. (E) HEK293 T, HuH7, and HepG2 cells were transfected with the PX458 plasmid and treated with AR7 (20 µM) for 24 h after 48 h of transfection, and the levels of Cas9 and LAMP2A proteins were detected by Western blotting. (F) The binding of Cas9 and HSC70 in HeLa-Cas9 cells was detected by Co-IP using HSC70 antibody (anti-HSC70) and (G) Flag antibody (anti-Flag), respectively. (H) The distribution of Cas9 protein (green) and LAMP2A protein (red) in HeLa-Cas9 cells was detected by confocal microscopy. The cells were permeabilized with a gentle cell permeabilizer, Tween-20, which did not permeabilize the nuclear membrane. (I) Four KFERQ-like motifs were predicted in the Cas9 protein. (J) HeLa-Cas9 cells were transfected with si-NC, si-HSC70-1, or si-HSC70-2, and the levels of Cas9 and HSC70 proteins were detected by Western blotting. (K) HuH7 and HepG2 cells were co-transfected with the PX458 plasmid and si-NC, si-HSC70-1, or si-HSC70-2, and the levels of Cas9 and HSC70 proteins were detected by Western blotting. (L) HSC70 expression plasmid (PCDH-HSC70-HA) was transfected into HeLa-Cas9 cells, and the levels of Cas9 and HSC70 proteins were detected by Western blotting. α-tubulin protein was used as the internal control for Western blotting. Data are presented as mean ± SD of three independent experiments. ***P < 0.001, two-tailed Student’s *t* test.

To uncover the degradation mechanism of Cas9 protein in cells, we first treated HeLa cells stably expressing Cas9 (HeLa-Cas9 cells) with a UPS- and ALP-specific inhibitor, respectively. The results showed that there was no apparent change in the level of Cas9 protein after the treatment of MG132, a UPS-specific inhibitor (supplementary Figure S1A), whereas the level of Cas9 protein increased after the treatment of BafA1, an ALP-specific inhibitor (Figure 1(A) and supplementary Figure S1B). The same phenomenon was also observed in HEK293 T, HuH7, and HepG2 cells transiently transfected with Cas9 expression plasmid (PX458) (Figure 1(B) and supplementary Figures S1C and S1D), suggesting that Cas9 protein was primarily degraded through the ALP pathway.

Next, HeLa-Cas9 cells and the PX458 plasmid-transfected HEK293 T, HuH7, and HepG2 cells were treated with LY294002, a macroautophagy-specific activator [35]. As a result, there was no change in the level of Cas9 protein when the level of SQSTM1/p62 protein, a substrate protein of macroautophagy, decreased by treating with LY294002 (supplementary Figures S1E and S1F). There was also no apparent change in the level of Cas9 protein after knocking out *SQSTM1/p62*, *ATG3*, or *ATG5* gene (supplementary Figures S1G–S1I). Due to the lack of a microautophagy-specific inhibitor or activator, HeLa-Cas9 cells were next treated with 7-Chloro-3-(4-methylphenyl)-2H-1,4-benzoxazine (AR7), a CMA-specific activator that promotes CMA by upregulating the transcription of *the LAMP2A* gene encoding a lysosomal membrane receptor–LAMP2A protein [36]. As a result, the level of Cas9 protein significantly decreased when CMA was activated by AR7 (Figures 1(C,D), and Supplementary Figures S2A–S2C). The same phenomenon was also found in HEK293 T, HuH7, and HepG2 cells (Figure 1(E) and supplementary Figures S2D and S2E). After knocking out the *LAMP2A* gene, the level of Cas9 protein increased (supplementary Figures S2F and S2G), and there was no apparent change in the level of Cas9 protein under AR7 treatment (supplementary Figure S2H). Furthermore, when the CMA-lysosome pathway was induced by starvation, the level of Cas9 protein also decreased with the increase of LAMP2A protein level (supplementary Figures S2I–S2K). The above results suggest that Cas9 protein is primarily degraded through the CMA-lysosome pathway.

Since most proteins degraded through the CMA pathway are mediated by molecular chaperone HSC70 and contain KFERQ-like motifs that can be bound by HSC70 protein [27], we first performed a Co-IP assay to detect the binding activity of Cas9 and HSC70 protein. The result showed that Cas9 protein could be pulled down by HSC70 (Figure 1(F)). In turn, HSC70 protein could also be pulled down by Cas9 (Figure 1(G)). Meanwhile, we found that Cas9 protein could co-localize with HSC70 and LAMP2A proteins in cells (Figure 1(H) and supplementary Figure S3A). Further, we analysed the amino acid sequence of the Cas9 protein. As a result, four KFERQ-like motifs were observed in Cas9 protein, including IRDKQ located at amino acid (aa) 670–674 in the REC3 domain, QILKE located at aa 794–798 and QRKFD located at aa 894–898 in the HNH domain, and RKDFQ located at aa 967–971 in the RuvC-III domain (Figure 1(I)). Furthermore, the level of the Cas9 protein increased when the endogenous HSC70 expression was knocked down (Figures 1(J,K), and supplementary Figures S3B–S3E). In turn, overexpressing HSC70 decreased the level of the Cas9 protein (Figure 1(L) and supplementary Figures S3F–S3H).

We and other groups have found that the Cas9 protein can be released through exosomes [30,37–39]. The biogenesis of exosomes is composed of endosomal sorting complex required for transport (ESCRT) dependent and independent pathways. ESCRT-0 component-hepatocyte growth factor-regulated tyrosine kinase substrate (HRS) usually participates in the degradation of target protein through ALP [40,41]; thus, we speculated that HRS might also be involved in the degradation of Cas9 protein. As shown in supplementary Figures S4A–S4C, the level of Cas9 protein in HeLa-Cas9 cells was significantly decreased by knocking down HRS expression, and there was no effect on the level of the Cas9 mRNA (supplementary Figure S4D). In turn, overexpressing HRS increased the level of the Cas9 protein (supplementary Figures S4E and S4F). The same phenomenon was also found in the PX458 plasmid-transfected HuH7 cells (supplementary Figures S4G–S4J). However, the level of the Cas9 protein in exosomes did not change when the release of exosomes was inhibited by knocking down TSG101 or Alix expression (supplementary Figures S4K and S4L), indicating that the Cas9 protein was mainly secreted by the ESCRT-independent pathway. There was no effect of HRS on gRNA level (supplementary Figure S4M). Further, we found that the stability of Cas9 protein was significantly decreased by knocking down HRS expression (supplementary Figures S5A and S5B). The Cas9 protein can be pulled down by HRS (supplementary Figure S5C), and the HRS protein can also be pulled down by Cas9 (supplementary Figure S5D). Furthermore, the amount of the Cas9 protein pulled down by HSC70 decreased, but the amount of the HRS protein pulled down by HSC70 increased when HRS was overexpressed (supplementary Figure S5E). Consistently, the amount of the HSC70 protein pulled down by Cas9 also decreased, but the amount of the HRS protein pulled down by Cas9 also increased when HRS was overexpressed (supplementary Figure S5F). These results suggest that HRS can stabilize Cas9 protein by competing with HSC70 to bind to Cas9 and subsequently inhibiting its degradation *via* CMA. These findings further confirm that the Cas9 protein is primarily degraded through the HSC70-mediated CMA-lysosome pathway.

### KFERQ-like motifs at aa 670–674 and aa 894–898 mediate Cas9 degradation

**Figure 2.**
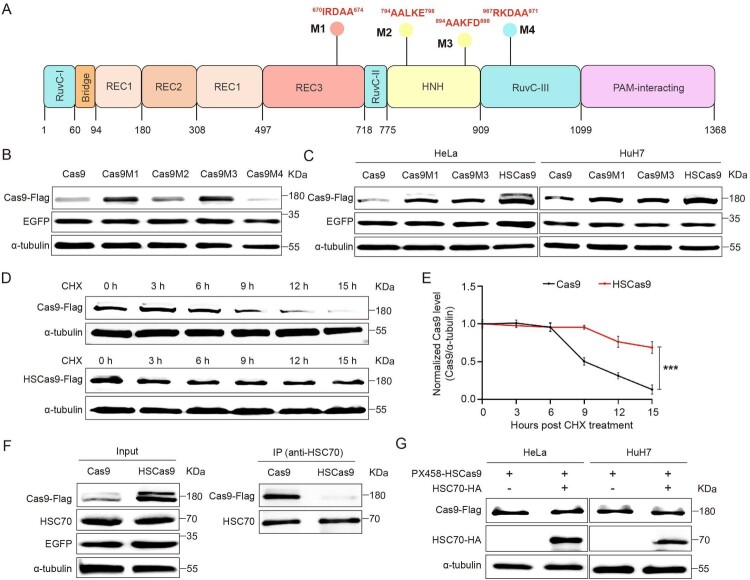
Screening the HSCas9 protein that can antagonize HSC70-mediated CMA degradation. (A) The mutant sequences of KFERQ-like motifs. (B) The PX458, PX458-Cas9M1, PX458-Cas9M2, PX458-Cas9M3, or PX458-Cas9M4 plasmid was transfected into HeLa cells, and the levels of Cas9 and EGFP proteins were detected by Western blotting. (C) The PX458, PX458-Cas9M1, PX458-Cas9M3, or PX458-Cas9M1 + 3 (HSCas9) plasmid was transfected into HeLa and HuH7 cells, and the levels of Cas9 and EGFP proteins were detected by Western blotting. (D) The HeLa cells were transfected with PX458 or PX458-HSCas9 plasmid and treated with Cycloheximide (CHX) for 0, 3, 6, 9, 12, and 15 h after 72 h of transfection. The level of Cas9 protein was detected by Western blotting. (E) The relative level of Cas9 protein was quantified by ImageJ software. (F) The PX458 or PX458-HSCas9 plasmid was transfected into HeLa cells, and Co-IP was performed with anti-HSC70. (G) The PX458-HSCas9 and PCDH-HSC70-HA plasmids were co-transfected into HeLa and HuH7 cells, and the levels of Cas9 and HSC70 proteins were detected by Western blotting. α-tubulin was used as the internal control for Western blotting. Data are presented as mean ± SD of three independent experiments. ***P < 0.001, two-tailed Student’s *t* test.

Further, we explored which KFERQ-like motif of the Cas9 protein mediated its self-degradation. Firstly, we constructed the Cas9 proteins with each KFERQ-like motif mutation, including IRDAA located at aa 670–674 (Cas9M1), AALKE located at aa 794–798 (Cas9M2), AAKFD located at aa 894–898 (Cas9M3), and RKDAA located at aa 967–971 (Cas9M4) (Figure 2(A)). As shown in Figure 2(B) and Supplementary Figure S6A, the levels of Cas9M1 and Cas9M3 proteins were higher than those of the wild-type Cas9 protein. Next, we constructed the IRDAA and AAKFD motifs double-mutated Cas9 (HSCas9) protein and found that its protein level further increased (Figure 2(C) and supplementary Figure S6B), and its stability was significantly higher than that of the wild-type Cas9 protein (Figures 2(D,E), supplementary Figures S6C and S6D). There was almost no binding ability between HSC70 and HSCas9 (Figure 2(F)). Moreover, there was no apparent change in the level of the HSCas9 protein when HSC70 was overexpressed (Figure 2(G)) and the CMA-lysosome pathway was induced by starvation (supplementary Figure S6E). These results suggest that HSCas9 can potentially antagonize HSC70-mediated CMA degradation.

### HSCas9 promotes the CRISPR/Cas9 system to destroy the HBV genome *in vitro*

**Figure 3.**
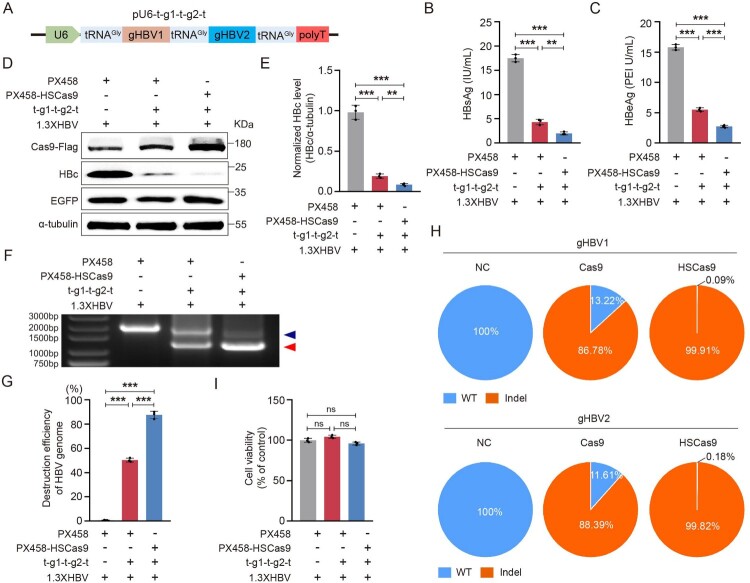
HSCas9 promoted the CRISPR/Cas9 system to destroy the HBV genome. (A) The illustration of the pU6-tRNA^Gly^-gHBV1-tRNA^Gly^-gHBV2-tRNA^Gly^ (pU6-t-g1-t-g2-t) plasmid. The pBB4.5–1.3 × HBV, PX458, or PX458-HSCas9 plasmid, and pU6-t-g1-t-g2-t plasmid or vector control were co-transfected into HuH7 cells. The levels of (B) HBsAg and (C) HBeAg in the cell culture supernatants were detected by chemiluminescence immunoassay. (D) The levels of Cas9, EGFP, and HBc proteins were detected by Western blotting, and α-tubulin was used as the internal control. (E) The relative level of HBc protein was quantified by ImageJ software. (F) The levels of the complete HBV genome (indicated by blue arrow head) and the short HBV genome formed by the two gHBVs-mediated cleavages (indicated by red arrow head) were detected by PCR with the primers outside the two cleavage sites, and the PCR products were detected by agarose gel electrophoresis. (G) The destruction efficiency of the HBV genome was evaluated by the ratio of short HBV genome to total HBV genome (complete HBV genome + short HBV genome), which was quantified by ImageJ software. (H) The total PCR products were sequenced by next-generation sequencing, and the indel variant rate was calculated as the number of indel variant reads divided by the total number of reads at the cleavage site. (I) The cell viability was detected by the CCK-8 assay. Data are presented as mean ± SD of three independent experiments. **P < 0.01, ***P < 0.001, ns = no statistical significance, two-tailed Student’s *t* test.

It has been reported that transfer RNA (tRNA) can be used to express multiple guide RNAs (gRNAs) [42]. To explore the ability of HSCas9 in destroying the HBV genome, we designed a tRNA^Gly^-gHBV1-tRNA^Gly^-gHBV2-tRNA^Gly^ tandem array using natural tRNA carrying glycine (tRNA^Gly^) and two HBV-specific gRNAs (gHBV1 and gHBV2) that can efficiently destroy the HBV genome in our previous study [34] (Figure 3(A)). The ability of HSCas9 to reduce the levels of HBsAg and HBeAg was significantly higher than that of wild-type Cas9 (Figures 3(B,C)). Due to the inability of eukaryotic ribosomes to insert a peptide bond between the Gly (G) and Pro (P) residues in the 2A peptide of Thosea asigna virus capsid (T2A) located between the coding sequences of Cas9 and enhanced green fluorescent protein (EGFP) in PX458 plasmid, the isolated Cas9 and EGFP proteins will be generated simultaneously [4]. To exclude differences in transfection efficiency, we simultaneously detected the levels of HBc and EGFP proteins in cells, and found that the ability of HSCas9 to reduce the level of HBc was significantly higher than that of wild-type Cas9 (Figures 3(D,E)). Further, we found that the level of short HBV genome formed by the two gHBVs/HSCas9-mediated cleavages was significantly higher than that of the two gHBVs/wild-type Cas9-mediated cleavages (Figures 3(F,G)), and the indel variant rate induced by each gHBV/HSCas9 was higher than that induced by each gHBV/wild-type Cas9 (Figure 3(H)). There was no obvious effect of HSCas9 on cell viability (Figure 3(I)). Meanwhile, we additionally constructed the other tRNA^Gly^-gHBV3-tRNA^Gly^-gHBV4-tRNA^Gly^ (t-g3-t-g4-t) tandem array carrying another two HBV-specific gRNAs (gHBV3 and gHBV4) that can also efficiently destroy the HBV genome in our previous study [34]. As shown in supplementary Figures S7A–S7D, the ability of HSCas9 to inhibit HBV replication was also significantly higher than that of wild-type Cas9. Moreover, we found that HRS could also promote the CRISPR/Cas9 system to inhibit HBV replication by promoting the destruction of HBV genome (supplementary Figures S8A–S8J).

As off-target effects are one of the major concerns for the CRISPR/Cas9 system, we next analysed whether HSCas9 increased off-target effects. After predicting the potential off-target sites of gHBV1 using the Cas-OFFinder tool [33], the next-generation sequencing was performed on the top three predicted off-target sites (supplementary Tables S4 and S5). The results showed that the off-target effects of both wild-type Cas9 and HSCas9 were very low, and HSCas9 did not increase off-target effects when compared to wild-type Cas9 (Supplementary Table S6).

Further, we explored whether the mutated KFERQ-like motifs affected the nuclease activity of the Cas9 protein. To exclude the influence of different Cas9 protein levels as much as possible, the transfection amount of wild-type Cas9, Cas9M1, Cas9M3 or HSCas9 expression plasmid was much higher than that of the 1.3×HBV plasmid. The result showed that the mutated KFERQ-like motifs of Cas9 protein did not affect the nuclease activity of Cas9 protein (supplementary Figures S9A–S9C), suggesting that HSCas9 promoted the CRISPR/Cas9 system to destroy HBV genome due to its higher stability.

**Figure 4.**
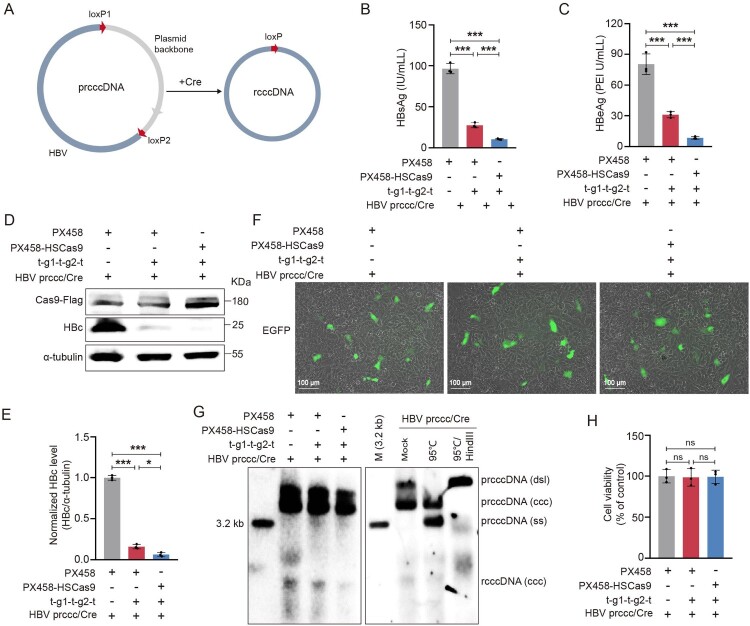
HSCas9 promoted the CRISPR/Cas9 system to destroy HBV cccDNA. (A) A schematic illustration of Cre/loxP-mediated rcccDNA production. HepG2 cells were co-transfected with the HBV prccc/Cre plasmid, PX458 or PX458-HSCas9 plasmid, and pU6-t-g1-t-g2-t plasmid or vector control. The levels of (B) HBsAg and (C) HBeAg in the cell culture supernatants were detected by chemiluminescence immunoassay. (D) The levels of Cas9 and HBc proteins were detected by Western blotting, and α-tubulin was used as the internal control. (E) The relative level of HBc protein was quantified by ImageJ software. (F) The level of EGFP protein was detected by immunofluorescence. (G) The level of HBV rcccDNA was detected by Southern blotting. (H) The cell viability was detected by CCK-8 assay. Data are presented as mean ± SD of three independent experiments. *p < 0.05, ***P < 0.001, ns = no statistical significance, two-tailed Student’s *t* test.

Next, the ability of HSCas9 to destroy HBV cccDNA was evaluated by a Cre/loxP-based recombinant HBV cccDNA (rcccDNA) system [43,44]. In this system, a monomeric HBV genome was engineered into a precursor plasmid (prcccDNA), which was excised via Cre/loxP-mediated DNA recombination to form a 3.3 kb rcccDNA (Figure 4(A)). As shown in Figures 4(B–E), the ability of HSCas9 to reduce the levels of HBsAg, HBeAg, and HBc was significantly higher than that of wild-type Cas9 in the Cre/loxP-based rcccDNA system. The level of EGFP protein in each group was used to exclude the difference in transfection efficiency (Figure 4(F)). Meanwhile, we found that the ability of HSCas9 to reduce the level of rcccDNA was significantly higher than that of wild-type Cas9 (Figure 4(G)), and there was no obvious effect of HSCas9 on cell viability (Figure 4(H)). These results indicate that HSCas9 promotes the CRISPR/Cas9 system to destroy HBV cccDNA.

**Figure 5.**
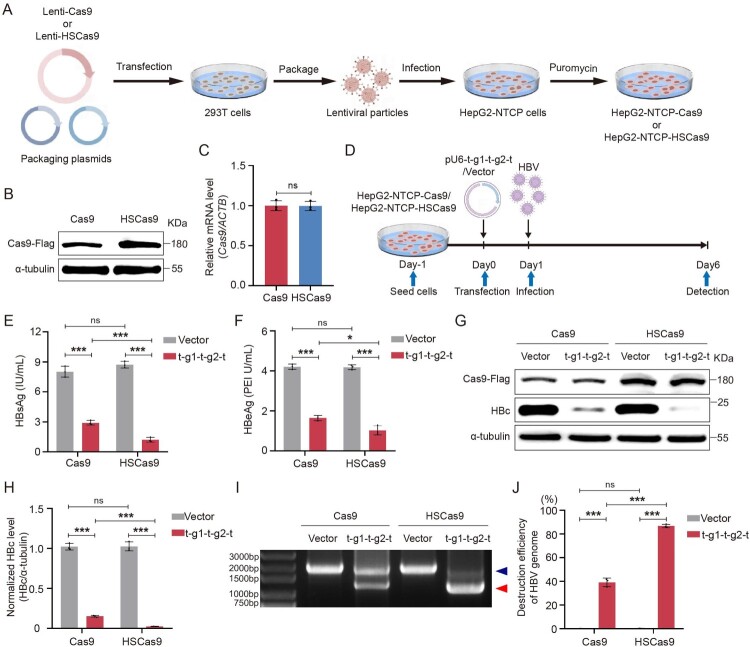
HSCas9 promoted the CRISPR/Cas9 system to clear HBV infection. (A) The schematic illustration of the construction for HepG2-NTCP-Cas9 and HepG2-NTCP-HSCas9 cells. (B) The levels of the Cas9 protein in HepG2-NTCP-Cas9 cells and the HSCas9 protein in HepG2-NTCP-HSCas9 cells were detected by Western blotting. (C) The mRNA levels of *Cas9* in HepG2-NTCP-Cas9 cells and *HSCas9* in HepG2-NTCP-HSCas9 cells were detected by RT-qPCR, and *ACTB* mRNA was used as the internal control. (D) HepG2-NTCP-Cas9 and HepG2-NTCP-HSCas9 cells were transfected with pU6-t-g1-t-g2-t plasmid or vector control, and then were infected with HBV (500 geq/cell). At 5 days post-infection, the levels of (E) HBsAg and (F) HBeAg in the culture supernatants were detected by chemiluminescence immunoassays. (G) The levels of Cas9 and HBc proteins were detected by Western blotting. (H) The relative level of HBc protein was quantified by ImageJ software. (I) The levels of the complete HBV genome (indicated by blue arrow head) and the short HBV genome formed by the two gHBVs-mediated cleavages (indicated by red arrow head) were detected by PCR with the primers outside the two cleavage sites, and the PCR products were detected by agarose gel electrophoresis. (J) The destruction efficiency of the HBV genome was evaluated by the ratio of short HBV genome to total HBV genome (complete HBV genome + short HBV genome), which was quantified by ImageJ software. α-tubulin was used as the internal control for Western blotting. Data are presented as mean ± SD of three independent experiments. *p < 0.05, ***P < 0.001, ns = no statistical significance, two-tailed Student’s *t* test.

To further confirm the ability of HSCas9 to clear HBV infection, HepG2-NTCP cells stably expressing Cas9 (HepG2-NTCP-Cas9 cells) or HSCas9 (HepG2-NTCP-HSCas9 cells) were constructed (Figure 5(A)). As shown in Figure 5(B) and supplementary Figure S10A, the level of HSCas9 protein was higher than that of Cas9 protein, but the mRNA levels of *HSCas9* and *Cas9* were equivalent between the HepG2-NTCP-HSCas9 and HepG2-NTCP-Cas9 cells (Figure 5(C)), which further confirmed that the stability of HSCas9 was higher than that of wild-type Cas9. Next, both cells were transfected with pU6-t-g1-t-g2-t plasmid or vector control, and then were infected with HBV (500 geq/cell) collected and concentrated from HepAD38 cells (Figure 5(D)). The results revealed that the levels of HBsAg and HBeAg in the culture supernatants of HepG2-NTCP-HSCas9 cells were significantly lower than those of HepG2-NTCP-Cas9 cells (Figures 5(E,F)). The level of HBc in HepG2-NTCP-HSCas9 cells was also significantly lower than that of HepG2-NTCP-Cas9 cells (Figures 5(G,H)). Furthermore, the destruction efficiency of HBV genome in HepG2-NTCP-HSCas9 cells was significantly higher than that of HepG2-NTCP-Cas9 cells (Figures 5(I,J)), and the level of complete cccDNA in HepG2-NTCP-HSCas9 cells was significantly lower than that of HepG2-NTCP-Cas9 cells (supplementary Figure S10B), which further confirmed that the ability of HSCas9 to destroy cccDNA was significantly higher than that of wild-type Cas9. There was also no obvious effect of HSCas9 on cell viability (supplementary Figure S10C). These results suggest that HSCas9 promotes the CRISPR/Cas9 system to clear HBV infection.

Furthermore, we compared the efficacy of HSCas9/gHBV and entecavir (ETV) in inhibiting HBV replication. As shown in supplementary Figures S11A–S11E, the ability of HSCas9/gHBV to inhibit HBV replication was significantly higher than that of ETV, suggesting that HSCas9/gHBV has an advantage in clearing HBV infection.

### HSCas9 promotes the CRISPR/Cas9 system to destroy the HBV genome *in vivo*

**Figure 6.**
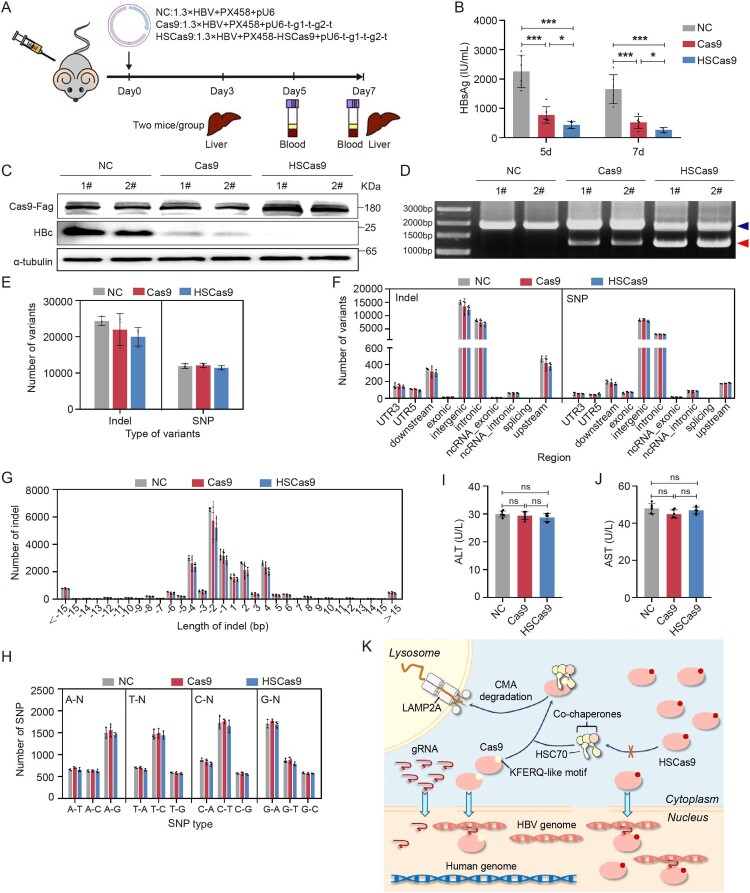
HSCas9 promoted the CRISPR/Cas9 system to destroy the HBV genome in mice. (A) A schematic illustration of the design of the hydrodynamic injection mouse model. (B) The levels of HBsAg in sera were detected by chemiluminescence immunoassays at 5 and 7 days after hydrodynamic injection. At 3 days after hydrodynamic injection, (C) the levels of HBc protein in the liver tissues of two mice were detected by Western blotting, and (D) the levels of the complete HBV genome (indicated by blue arrow head) and the short HBV genome formed by the two gHBVs-mediated cleavages (indicated by red arrow head) were detected by PCR with the primers outsides two cleave sites, and the PCR products were detected by agarose gel electrophoresis. At 7 days after hydrodynamic injection, genomic DNA was extracted from the liver tissues of three mice in each group, followed by whole genome sequencing. (E) The total number of SNPs and indel variants. (F) The number of SNPs and indel variants at different regions of the genome. (G) The number of indel variants at different lengths. (H) The number of SNPs of different types. The levels of (I) ALT and (J) AST in sera were detected by ELISA. (K) The graphical abstract on the role of CMA in the destruction of HBV genome by the CRISPR/Cas9 system. Data are presented as mean ± SD of three independent experiments. *p < 0.05, ***P < 0.001, ns = no statistical significance, two-tailed Student’s *t* test.

To explore the ability of HSCas9 to destroy HBV genome *in vivo*, the hydrodynamic injection experiment was carried out in 24 male C57BL/6 mice (5 weeks old), which were randomly divided into negative control (NC), Cas9, and HSCas9 groups (Figure 6(A)). The results revealed that HSCas9 promoted the CRISPR/Cas9 system to reduce the levels of HBsAg and HBc (Figures 6(B,C), supplementary Figure S12A), and also the CRISPR/Cas9 system to destroy HBV genome *in vivo* (Figure 6(D)).

To explore the off-target effects of CRISPR/Cas9 and CRISPR/HSCas9 systems *in vivo*, we performed whole-genome sequencing for genomic DNA extracted from liver tissues of 3 mice in each group. The results revealed that there were no significant differences in the numbers of SNP and indel variants among the three groups (Figures 6(E–H)), and no SNP and indel variants were found within 20 bp upstream and downstream of all potential off-target sites (supplementary Table S7), suggesting that HSCas9 did not increase off-target effects. To exclude the effects of cytotoxicity, haematoxylin–eosin (HE) staining of liver tissue was performed, and the levels of alanine aminotransferase (ALT) and aspartate aminotransferase (AST) in serum were detected. The results showed that both CRISPR/Cas9 and CRISPR/HSCas9 systems did not cause significant liver injury and inflammation (Figures 6(I,J), Supplementary Figure S12B).

## Discussion

Chronic hepatitis B (CHB), an important risk factor for cirrhosis and hepatocellular carcinoma, is still difficult to cure. Although the currently used antiviral drugs can effectively inhibit HBV replication, it is challenging to achieve a functional cure for CHB, and long-term therapy has been recommended [45]. The reason why HBV infection is difficult to eradicate is that HBV cccDNA is stably present in the nuclei of hepatocytes, and currently used antiviral drugs cannot directly destroy cccDNA. With the development of gene editing technology, it has become possible to directly destroy the viral genome. Due to the advantages of simplicity and efficiency, the CRISPR/Cas9 system is the most promising gene editing technology for clearing viral infection. Different from the human genome, the viral genome is constantly replicating in human cells. Therefore, a more stable CRISPR/Cas9 system is needed to destroy the continuously replenished HBV cccDNA.

This study found that Cas9 protein was primarily degraded through the HSC70-mediated CMA-lysosome pathway. After mutating KFERQ-like motifs of Cas9 protein, we found that the level of HSCas9 protein with double mutations in two KFERQ-like motifs located at aa 670–674 and aa 894–898 was not reduced by overexpressing HSC70, which resulted in the higher stability of HSCas9 compared to wild-type Cas9. In terms of mechanism, the binding ability between HSCas9 to HSC70 had almost disappeared, suggesting that the two KFERQ-like motifs located at aa 670–674 and aa 894–898 of Cas9 protein mainly mediated the recognition of HSC70.

Next, we found that the ability of HSCas9 to destroy the HBV genome and subsequently inhibit HBV replication was higher than that of wild-type Cas9. Since the mutant KFERQ-like motif located at aa 670–674 or aa 894–898 did not affect the nuclease activity of the Cas9 protein, it is suggested that HSCas9 did not enhance its ability to destroy the HBV genome by increasing its nuclease activity. Further, HSCas9 did not increase the off-target effects compared to that of wild-type Cas9 *in vitro* and *in vivo*, which might be due to the highly HBV-specific gRNAs. In addition, both CRISPR/Cas9 and CRISPR/HSCas9 systems did not exhibit apparent cytotoxicity *in vitro* and *in vivo*. Therefore, HSCas9 promoted the CRISPR/Cas9 system to destroy the HBV genome due to its higher stability. Consistently, HRS can also promote the CRISPR/Cas9 system to inhibit HBV replication by competing with HSC70 to bind to Cas9 and subsequently enhancing the stability of the Cas9 protein. These results suggest that antagonizing HSC70-mediated CMA degradation of the Cas9 protein can promote the CRISPR/Cas9 system to destroy the HBV genome. In view of this, the HSCas9 protein can promote the CRISPR/Cas9 system to clear the infections caused by HBV or other viruses. Moreover, the conserved KFERQ-like motifs are also present in the deactivated Cas9 from *Streptococcus pyogenes* (SpdCas9) that can be used to regulate gene expression [46], Cas9 from *Staphylococcus aureus* (SaCas9) that can be packaged into the commonly used adeno associated virus delivery systems [47], as well as Cas12a and Cas13a that can be used for nucleic acid detection (supplementary Figure S12C) [48,49]. By utilizing the strategy of this study, the corresponding highly stable Cas protein may also be developed, which may improve its ability of gene expression regulation or the sensitivity of *in situ* nucleic acid detection.

Cas9 can bind to HSC70 through its KFERQ-like motifs and is subsequently degraded through the CMA-lysosome pathway. The HSCas9 protein with the mutant KFERQ-like motifs located at aa 670–674 and aa 894–898 is highly stable by antagonizing the HSC70-mediated CMA degradation, which promotes the CRISPR/Cas9 system to clear HBV infection by destroying the HBV genome (Figure 6(K)). This study suggests that CMA can weaken the destructive ability of the CRISPR/Cas9 system to constantly replicate viral genomes, and provides a strategy to improve the ability of the CRISPR/Cas9 system to clear viral infections by antagonizing HSC70-mediated CMA degradation. However, compared to wild-type Cas9, HSCas9 may have a higher risk of off-target effects for those gRNAs with low specificity. Therefore, selecting highly specific gRNAs is crucial for using CRISPR/Cas9 technology. For gene editing of non-replicating cellular genomes, the use of HSCas9 can potentially reduce the cost of gene editing by lowering the amount of Cas9 protein or mRNA, thereby promoting the widespread application of the CRISPR/Cas9 system.

## Author contributions

ZL, YS, and HH contributed equally to this work. JW, ZL, YS, and HH conceived the project and designed the experiments. ZL, YS, HH, RC, ML, XY, and ZL performed experiments. JW, ZL, YS, and HH directed the data analysis. ZL, YS, and HH drafted the manuscript. JW and BML edited the manuscript. All authors approved the final manuscript.

## Supplementary Material

Supplementary materials.docx

## Data Availability

The original data of next-generation sequencing are uploaded to the Sequence Read Archive (SRA) database: PRJNA1194370. The original data of whole genome sequencing are uploaded to the SRA database: PRJNA1195388. The data that support the findings of this study are available from the corresponding author upon reasonable request.
