# Supplementary material for: Highly stable Cas9 promotes HBV genome destruction by antagonizing HSC70-mediated degradation
Source: Emerg Microbes Infect. 2025 Sep 4;14(1):2556728. doi: 10.1080/22221751.2025.2556728 (PMC12456051; doi:10.1080/22221751.2025.2556728)
Supplement: Supplementary materials.docx [file TEMI_A_2556728_SM4342.docx]

**Supplementary information**

Table of contents

Supplementary figures………………………………………………………………...2

Supplementary tables…………………………………………………….…….…......17

**Supplementary figures**


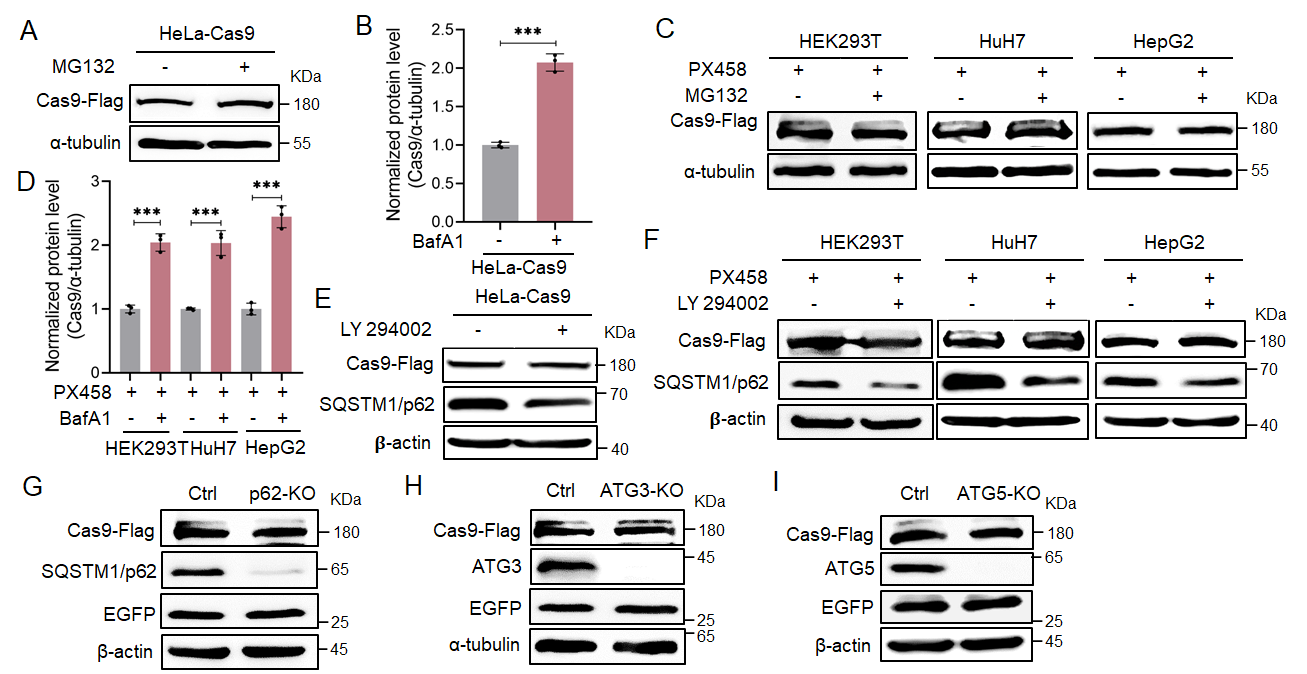


**Figure S1.** Cas9 protein was not degraded through UPS and macroautophagy pathways. (**A**) HeLa-Cas9 cells were treated with MG132 (10 μM) for 24 h, and the level of Cas9 protein was detected by Western blotting. (**B**) HeLa-Cas9 cells were treated with BafA1 (10 nM) for 12 h, and the relative level of Cas9 protein was quantified by ImageJ software. (**C**) HEK293T, HuH7, and HepG2 cells were transfected with Cas9 expression plasmid PX458 and treated with MG132 (10 μM) for 24 h after 48 h of transfection, and the level of Cas9 protein was detected by Western blotting. (**D**) HEK293T, HuH7 and HepG2 cells were transfected with Cas9 expression plasmid PX458 and treated with BafA1 (10 nM) for 12 h after 48 h of transfection, and the relative level of Cas9 protein was quantified by ImageJ software. (**E**) HeLa-Cas9 cells were treated with LY294002 (10 µM) for 24 h, and the levels of Cas9 and SQSTM1/p62 proteins were detected by Western blotting. (**F**) HEK293T, HuH7, and HepG2 cells were transfected with Cas9 expression plasmid PX458 and treated with LY294002 (10 µM) for 24 h after 48 h of transfection, and the levels of Cas9 and SQSTM1/p62 proteins were detected by Western blotting. (**G**) *SQSTM1/p62* knockout (KO) HEK293T cells or control cells were transfected with Cas9 expression plasmid PX458, and the levels of Cas9, SQSTM1/p62, and EGFP proteins were detected by Western blotting. (**H**) *ATG3* KO HEK293T cells or control cells were transfected with Cas9 expression plasmid PX458, and the levels of Cas9, ATG3, and EGFP proteins were detected by Western blotting. (**I**) *ATG5* KO HEK293T cells or control cells were transfected with Cas9 expression plasmid PX458, and the levels of Cas9, ATG5, and EGFP proteins were detected by Western blotting. β-actin or α-tubulin protein was used as the internal control for Western blotting. Data are presented as mean ± SD of three independent experiments. ****P*<0.001, two-tailed Student’s *t* test.


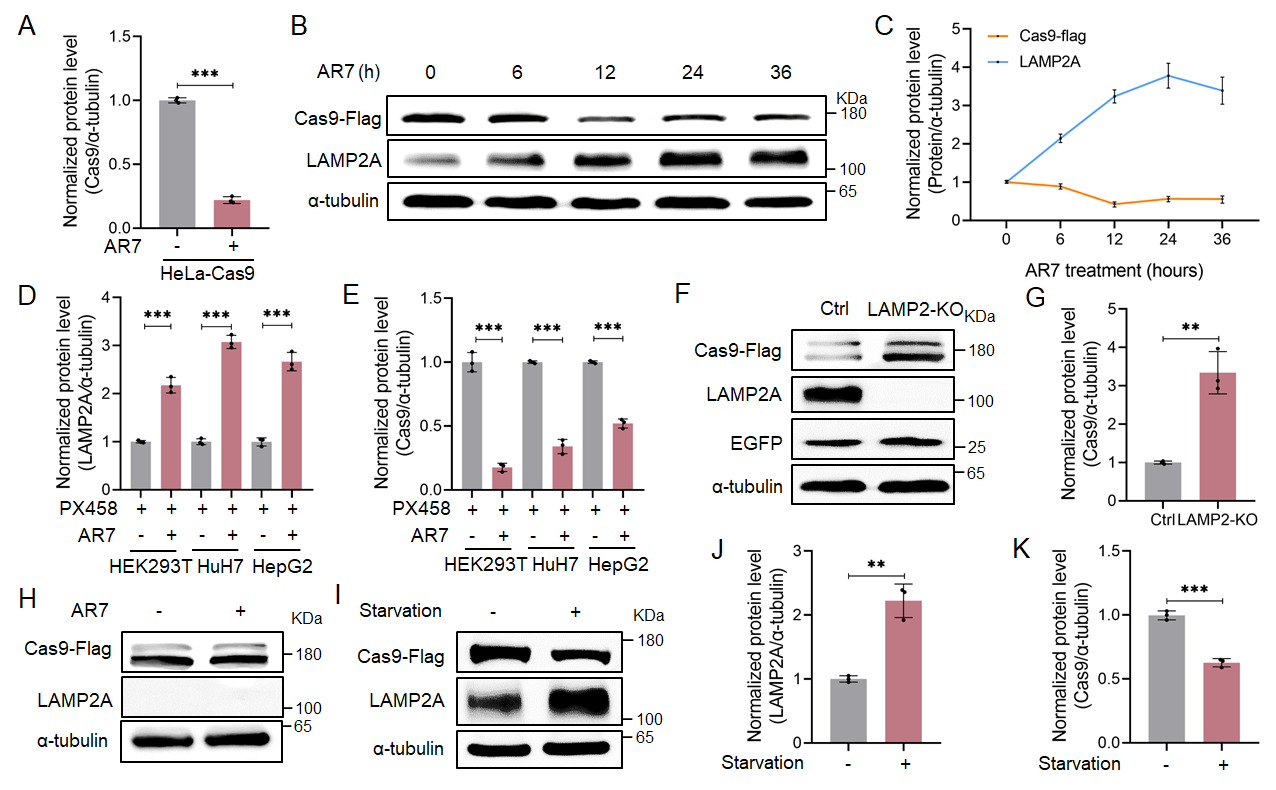


**Figure S2.** Cas9 protein was degraded through CMA-lysosome pathway. (**A**) HeLa-Cas9 cells were treated with AR7 (20 µM) for 24 h, and the relative level of Cas9 protein was quantified by ImageJ software. (**B**) HeLa-Cas9 cells were treated with AR7 (20 µM) for 0, 6, 12, 24, and 36 h, the levels of Cas9 and LAMP2A proteins were detected by Western blotting, and (**C**) the relative levels of Cas9 and LAMP2A proteins were quantified by ImageJ software. HEK293T, HuH7 and HepG2 cells were transfected with PX458 plasmid and treated with AR7 (20 µM) for 24 h after 48 h of transfection, and the relative levels of (**D**) LAMP2A and (**E**) Cas9 proteins were quantified by ImageJ software. (**F**) *LAMP2* KO HEK293T cells or control cells were transfected with Cas9 expression plasmid PX458, and the levels of Cas9, LAMP2A, and EGFP proteins were detected by Western blotting. (**G**) The relative level of Cas9 protein was quantified by ImageJ software. (**H**) *LAMP2* KO HEK293T cells were transfected with Cas9 expression plasmid PX458 and treated with AR7 (20 μM) for 24 h after 48 h of transfection, and the levels of Cas9 and LAMP2A proteins were detected by Western blotting. (**I**) HeLa-Cas9 cells were treated with starvation for 24 h, the levels of Cas9 and LAMP2A proteins were detected by Western blotting, and the relative levels of (**J**) LAMP2A and (**K**) Cas9 proteins were quantified by ImageJ software. α-tubulin protein was used as the internal control for Western blotting. Data are presented as mean ± SD of three independent experiments. ***P*<0.01, ****P*<0.001, two-tailed Student’s *t* test.


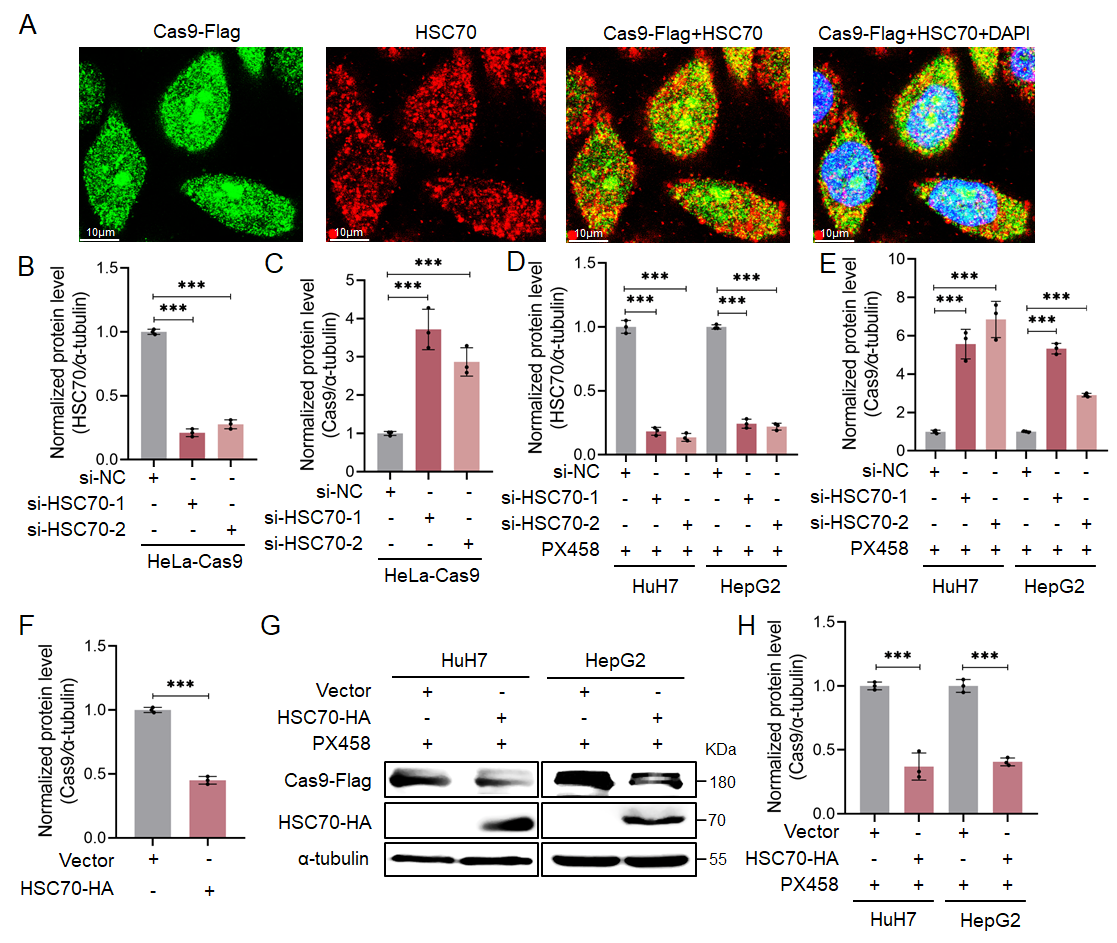


**Figure S3.** Cas9 protein was degraded through HSC70-mediated CMA-lysosome pathway. (**A**) The distributions of Cas9 protein (green) and HSC70 protein (red) in HeLa-Cas9 cells were detected using confocal microscopy. The cells were permeabilized with strong cell permeabilizer TritonX-100 which can also permeabilize the nuclear membrane. HeLa-Cas9 cells were transfected with si-NC, si-HSC70-1, or si-HSC70-2, and the relative levels of (**B**) HSC70 and (**C**) Cas9 proteins were quantified by ImageJ software. HuH7 and HepG2 cells were co-transfected with PX458 plasmid and si-NC, si-HSC70-1, or si-HSC70-2, and the relative levels of (**D**) HSC70 and (**E**) Cas9 proteins were quantified by ImageJ software. (**F**) HSC70 expression plasmid (PCDH-HSC70-HA) was transfected into HeLa-Cas9 cells, and the level of Cas9 protein was quantified by ImageJ software. **(G)** PX458 and PCDH-HSC70-HA or vector control were co-transfected into HuH7 and HepG2 cells, and the levels of Cas9 and HSC70 proteins were detected by Western blotting after 72 h of transfection. α-tubulin protein was used as the internal control. **(H)** The relative level of Cas9 protein was quantified by ImageJ software. Data are presented as mean ± SD of three independent experiments. ****P*<0.001, two-tailed Student’s *t* test.


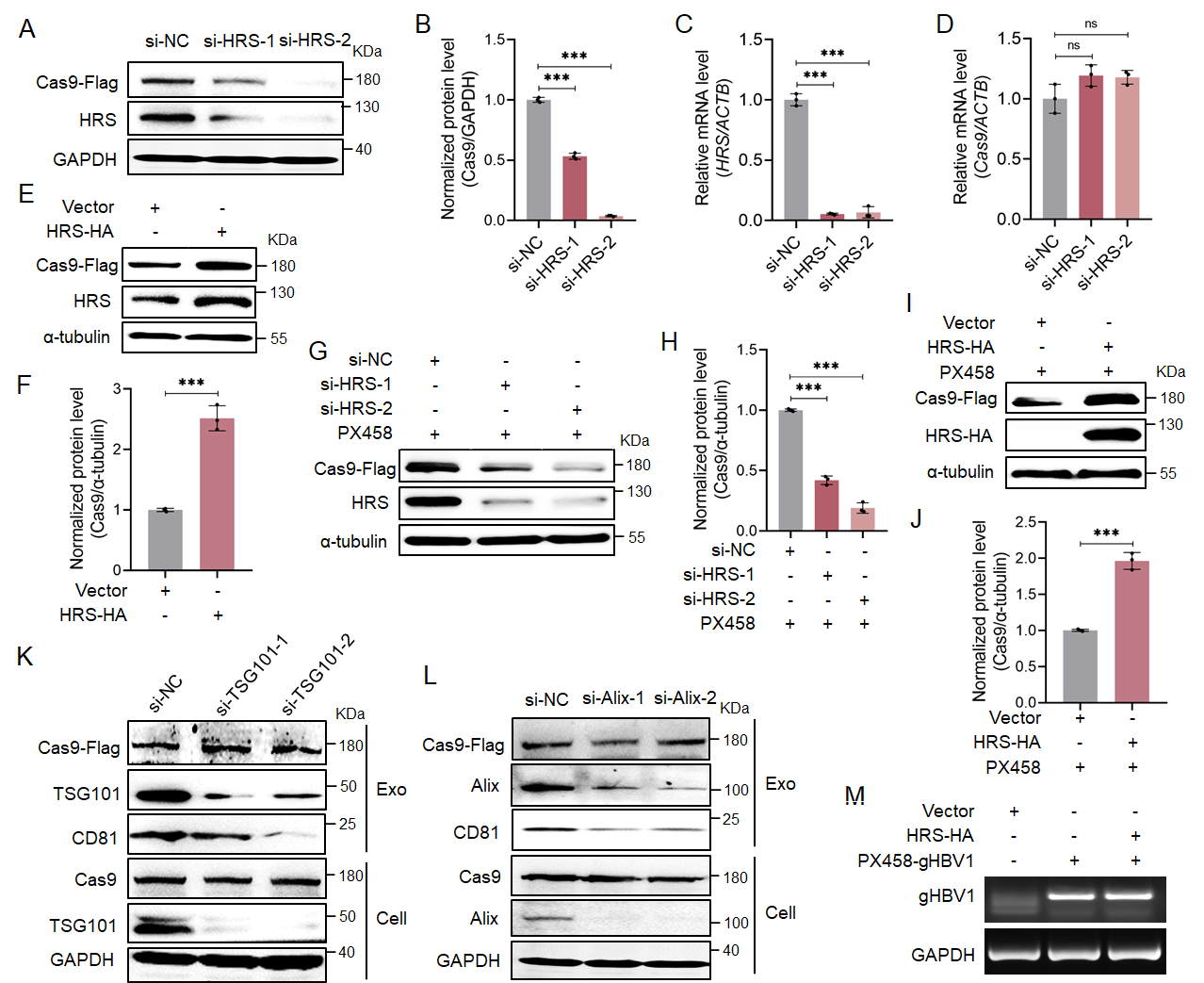


**Figure S4.** HRS upregulated the level of Cas9 protein. HeLa-Cas9 cells were transfected with HRS-specific siRNA-1 (si-HRS-1), si-HRS-2 or negative control scramble siRNA (si-NC), **(A)** the levels of Cas9 and HRS proteins were detected by Western blotting after 72 h of transfection, and (**B**) the relative level of Cas9 protein was quantified by ImageJ software. The relative levels of (**C**) *HRS* and (**D**) *Cas9* mRNA were detected by reverse-transcription-quantitative PCR (RT-qPCR). *ACTB* mRNA was used as the internal control. HRS expression plasmid PCDH-HRS-HA was transfected into HeLa-Cas9 cells, **(E)** the levels of Cas9 and HRS proteins were detected by Western blotting after 72h of transfection, and **(F)** the relative level of Cas9 protein was quantified by ImageJ software. PX458 plasmid and si-NC, si-HRS-1, or si-HRS-2 were co-transfected into HuH7 cells, **(G)** the levels of Cas9 and HRS proteins were detected by Western blotting after 72 h of transfection, and **(H)** the relative level of Cas9 protein was quantified by ImageJ software. PX458 and PCDH-HRS-HA plasmids or vector control (Vector) were co-transfected into HuH7 cells, **(I)** the levels of Cas9 and HRS proteins were detected by Western blotting after 72 h of transfection, and **(J)** the relative level of Cas9 protein was quantified by ImageJ software. **(K)** HeLa-Cas9 cells were transfected with TSG101-specific siRNA-1 (si-TSG101-1), si-TSG101-2, or si-NC, and the levels of Cas9 and TSG101 proteins in exosomes (Exo) and cells were detected by Western blotting after 72 h of transfection. **(L)** HeLa-Cas9 cells were transfected with Alix-specific siRNA-1 (si-Alix-1), si-Alix-2, or si-NC, and the levels of Cas9 and Alix proteins in exosomes (Exo) and cells were detected by Western blotting after 72 h of transfection. **(M)** The Cas9/HBV-specific (gHBV1) expression plasmid (PX458-gHBV1) and PCDH-HRS-HA or vector control (Vector) were co-transfected into HuH7 cells, and the level of gHBV1 in cells was detected by Semi-quantitative RT-PCR after 48 h of transfection. The PCR products were detected by 2.0% agarose gel electrophoresis. *GAPDH* mRNA was used as an internal control. GAPDH or α-tubulin protein was used as the internal control for Western blotting. Data are presented as mean ± SD of three independent experiments. ****P*<0.001, ns=no statistical significance, two-tailed Student’s *t* test.


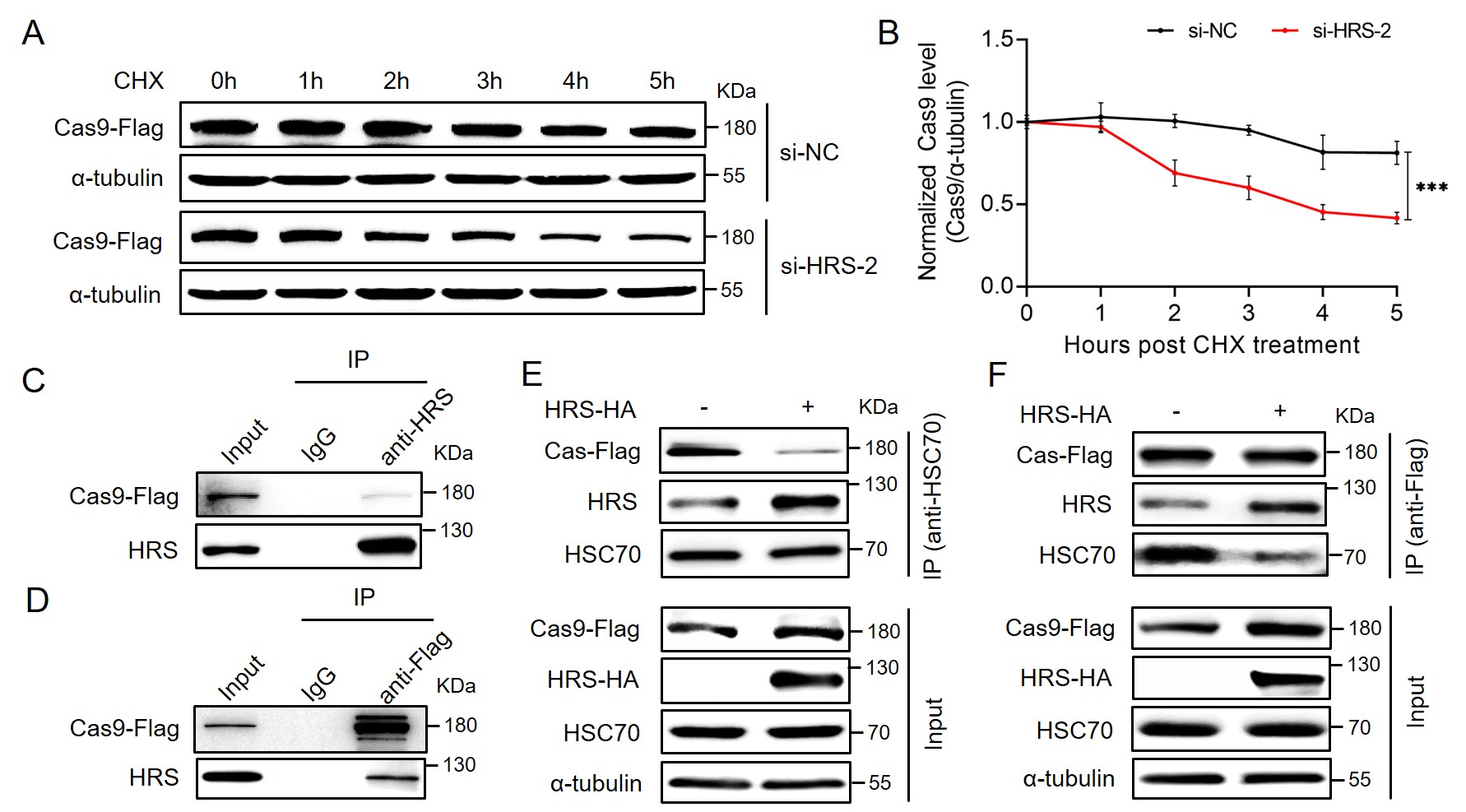


**Figure S5.** HRS stabilized Cas9 protein by competing with HSC70 to bind to Cas9. HeLa-Cas9 cells were transfected with si-NC or si-HRS-2 and treated with cycloheximide (CHX) for 0, 1, 2, 3, 4, and 5 h after 72 h of transfection, (**A**) the level of Cas9 protein was detected by Western blotting, and (**B**) the relative level of Cas9 protein was quantified by ImageJ software. (**C**) The binding of Cas9 and HRS in HeLa-Cas9 cells was detected by Co-IP using HRS antibody (anti-HRS). (**D**) The binding of Cas9 and HRS in HeLa-Cas9 cells was detected by Co-IP using anti-Flag. HeLa-Cas9 cells were transfected with PCDH-HRS-HA, and Co-IP assays were performed with (**E**) anti-HSC70 and (**F**) anti-Flag after 48 h of transfection. α-tubulin was used as the internal control for Western blotting. Data are presented as mean ± SD of three independent experiments. ****P* < 0.001, two-tailed Student’s *t* test.


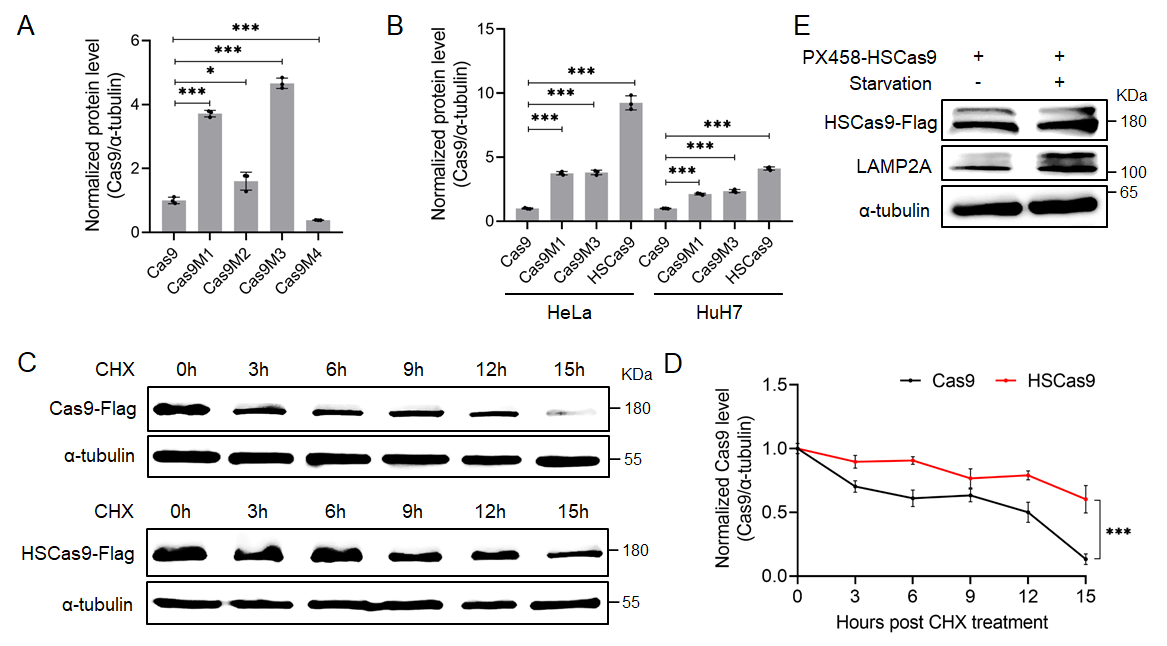


**Figure S6.** The stability of HSCas9 protein. (**A**) The PX458, PX458-Cas9M1, PX458-Cas9M2, PX458-Cas9M3, or PX458-Cas9M4 plasmid was transfected into HeLa cells, and the relative level of Cas9 protein was quantified by ImageJ software. (**B**) The PX458, PX458-Cas9M1, PX458-Cas9M3, or PX458-Cas9M1+3 (HSCas9) plasmid was transfected into HeLa and HuH7 cells, and the relative level of Cas9 protein was quantified by ImageJ software. HuH7 cells were transfected with PX458 or PX458-HSCas9 plasmid and treated with CHX for 0, 3, 6, 9, 12, and 15 h after 72 h of transfection, (**C**) the level of Cas9 protein was detected by Western blotting, and (**D**) the relative level of Cas9 protein was quantified by ImageJ software. (**E**) HeLa cells were transfected with PX458-HSCas9 plasmid and subjected to starvation treatment for 24 h after 48 h of transfection, and the levels of Cas9 and LAMP2A proteins were detected by Western blotting. α-tubulin protein was used as the internal control for Western blotting. Data are presented as mean ± SD of three independent experiments. **P*<0.05, ****P*<0.001, two-tailed Student’s *t* test.

**
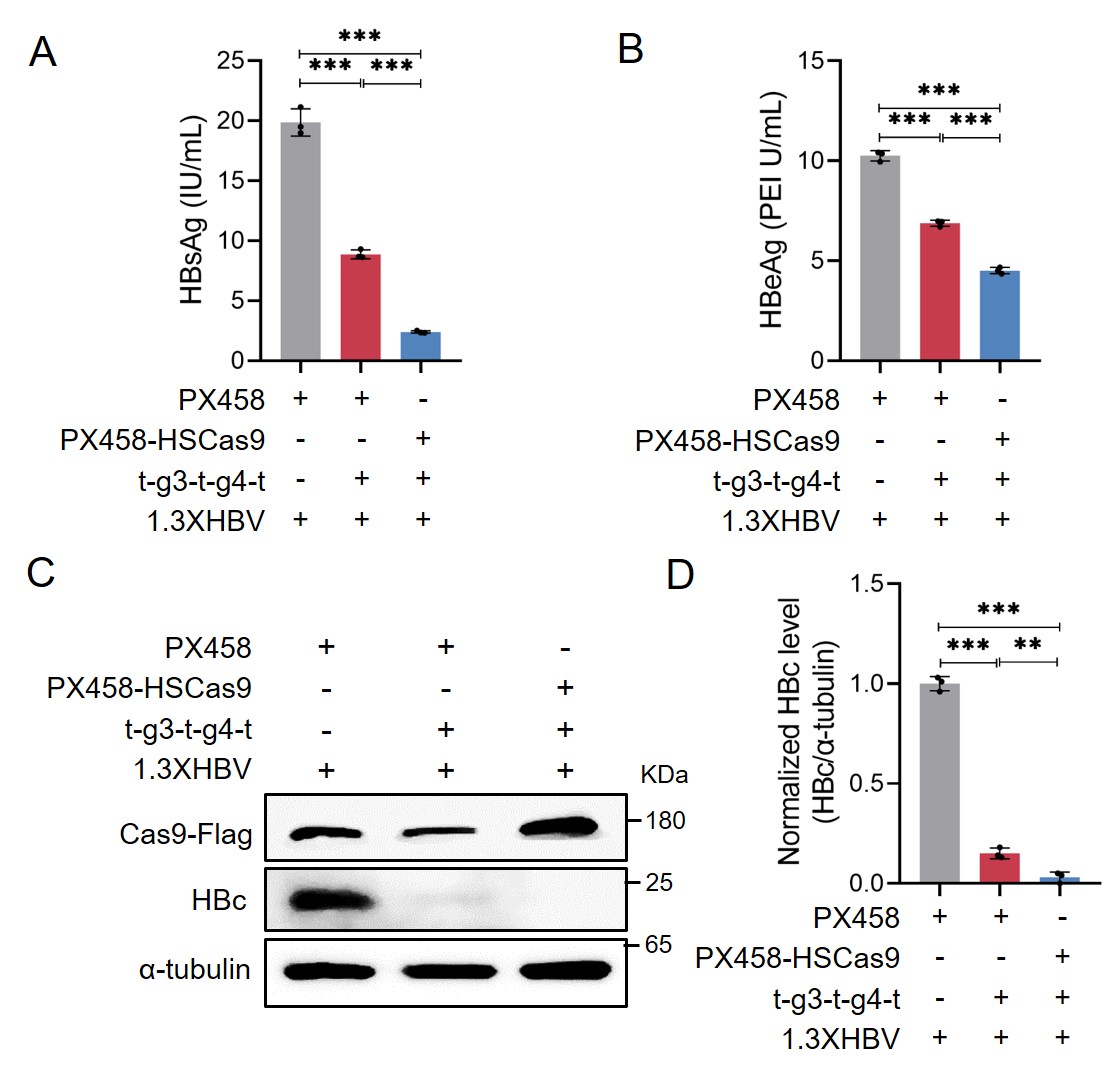
**

**Figure S7.** HSCas9 promoted the CRISPR/Cas9 system to inhibit HBV replication. The pBB4.5-1.3×HBV, PX458 or PX458-HSCas9 plasmid, and pU6-t-g3-t-g4-t plasmid or vector control were co-transfected into HuH7 cells. The levels of (**A**) HBsAg and (**B**) HBeAg in the cell culture supernatants were detected by chemiluminescence immunoassay. (**C**) The levels of Cas9 and HBc proteins were detected by Western blotting, and α-tubulin was used as the internal control. (**D**) The relative level of HBc protein was quantified by ImageJ software. Data are presented as mean ± SD of three independent experiments. ***P*<0.01, ****P*<0.001, two-tailed Student’s *t* test.


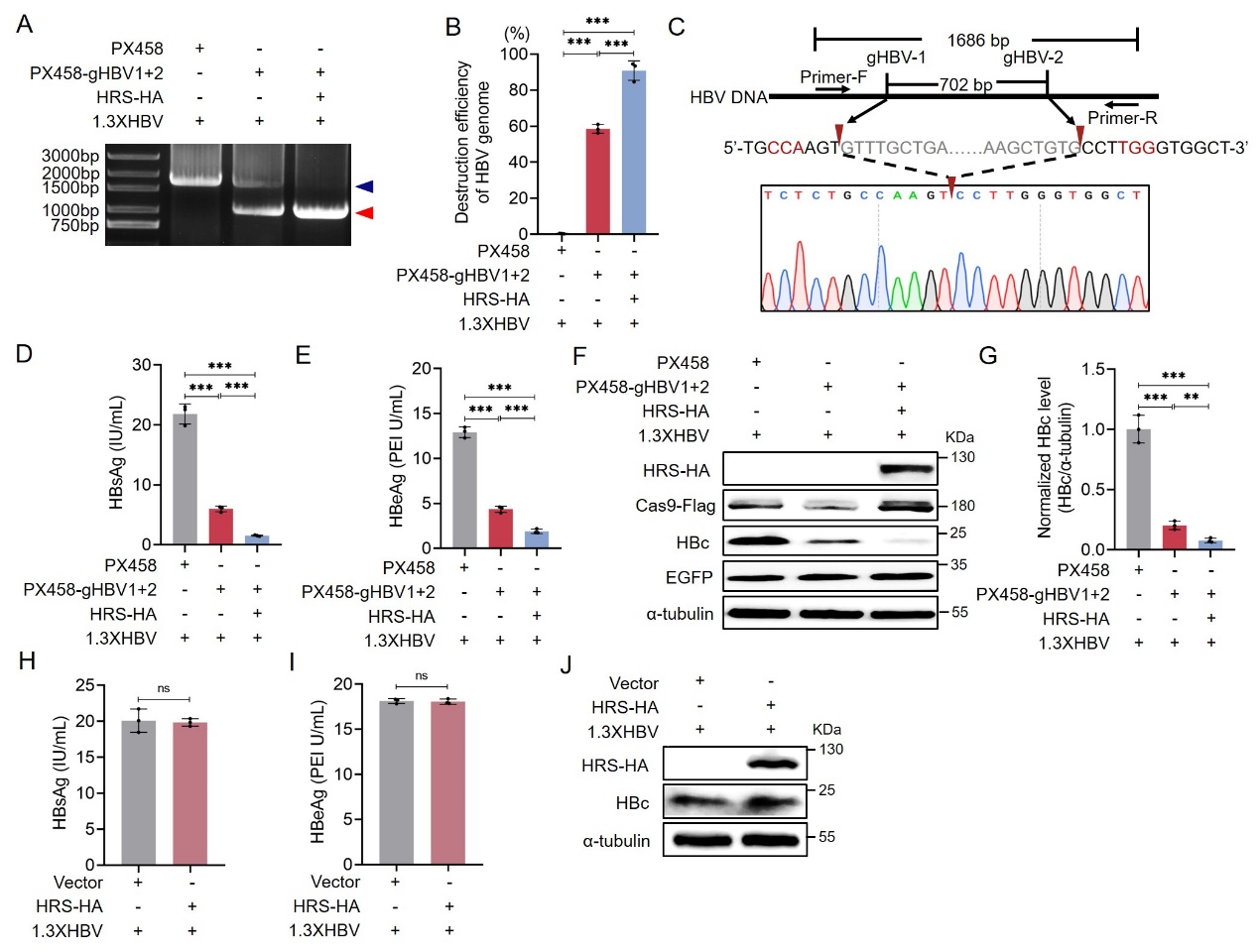


**Figure S8.** HRS promoted the CRISPR/Cas9 system to inhibit HBV replication. HuH7 cells were co-transfected with pBB4.5-1.3×HBV, PCDH-HRS-HA or vector control, and PX458-gHBV1+2 or PX458 plasmid. At 4 days post of transfection, the cells and supernatants were collected. (**A**) The levels of the complete HBV genome (indicated by blue arrow head) and the short HBV genome formed by the two gHBVs-mediated cleavages (indicated by red arrow head) were detected by PCR with the primers outsides two cleave sites, and the PCR products were detected by 1.5% agarose gel electrophoresis. (**B**) The destruction efficiency of HBV genome was evaluated by the ratio of short HBV genome to total HBV genome (complete HBV genome + short HBV genome), which was quantified by ImageJ software. (**C**) Sequencing analysis of the short HBV genome formed by two gHBVs-mediated cleavages. The levels of (**D**) HBsAg and (**E**) HBeAg in the cell culture supernatants were detected by chemiluminescence immunoassays. (**F**) The levels of Cas9-Flag, EGFP, HRS-HA, and HBc proteins were detected by Western blotting. (**G**) The relative level of HBc protein was quantified by ImageJ software. The pBB4.5-1.3×HBV and PCDH-HRS-HA plasmids were co-transfected into HuH7 cells, and the cells and supernatants were collected at 72 h post of transfection. The levels of (**H**) HBsAg and (**I**) HBeAg in the culture supernatants were detected by chemiluminescence immunoassays. (**J**) The levels of HRS-HA and HBc were detected by Western blotting, α-tubulin was used as the internal control for Western blotting. Data are presented as mean ± SD of three independent experiments. ***P*<0.01, ****P*<0.001, ns=no statistical significance, two-tailed Student’s *t* test.


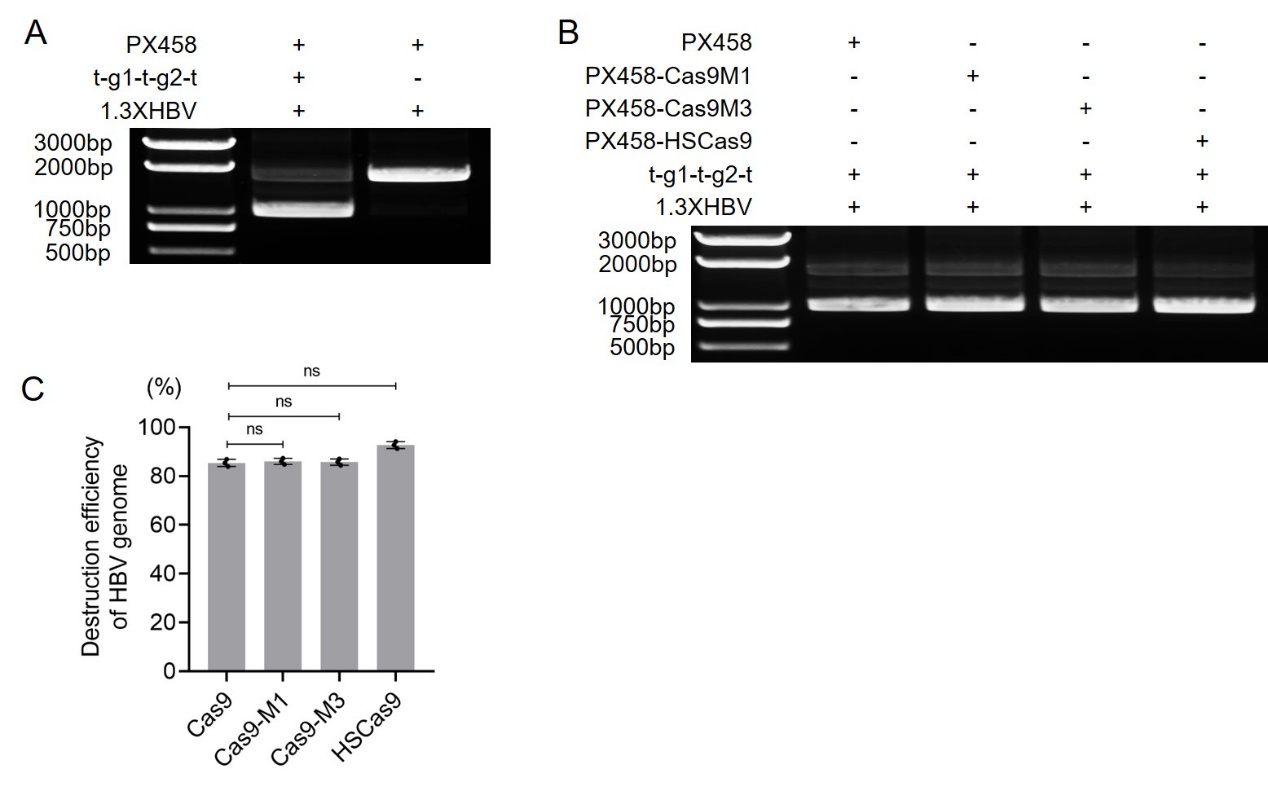


**Figure S9.** The nuclease activities of the wild-type and KFERQ-like motif mutant Cas9 proteins. (**A**) The pBB4.5-1.3×HBV, PX458, and pU6-t-g1-t-g2-t plasmid or vector control were co-transfected into HuH7 cells. At 72 h post of transfection, the levels of the complete HBV genome and the short HBV genome formed by the two gHBVs-mediated cleavages were detected by PCR, and the PCR products were detected by agarose gel electrophoresis. (**B**) The pBB4.5-1.3×HBV, PX458, each KFERQ-like motif mutant Cas9 expression plasmid, and pU6-t-g1-t-g2-t plasmids were co-transfected into HuH7 cells. At 72 h post of transfection, the levels of the complete HBV genome and the short HBV genome formed by the two gHBVs-mediated cleavages were detected by PCR, and the PCR products were detected by agarose gel electrophoresis. (**C**) The destruction efficiency of HBV genome was evaluated by the ratio of short HBV genome to total HBV genome (complete HBV genome + short HBV genome), which was quantified by ImageJ software. ns=no statistical significance, two-tailed Student’s *t* test.


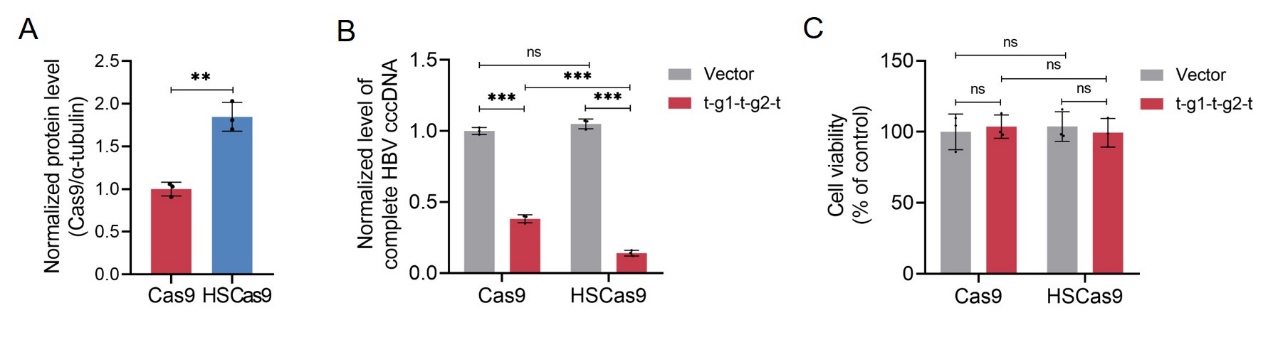


**Figure S10.** HSCas9 promoted the CRISPR/Cas9 system to clear HBV infection. (**A**) The relative levels of Cas9 protein in HepG2-NTCP-Cas9 cells and HSCas9 protein in HepG2-NTCP-HSCas9 cells were quantified by ImageJ software. The HepG2-NTCP-Cas9 and HepG2-NTCP-HSCas9 cells were transfected with pU6-t-g1-t-g2-t plasmid or vector control, and then were infected with HBV (500 geq/cell). At 5 days post of infection, (**B**) the level of complete HBV cccDNA was detected by qPCR. (**C**) The cell viability was detected by CCK-8 assay. Data are presented as mean ± SD of three independent experiments. ***P*<0.01, ****P*<0.001, ns=no statistical significance, two-tailed Student’s *t* test.

**
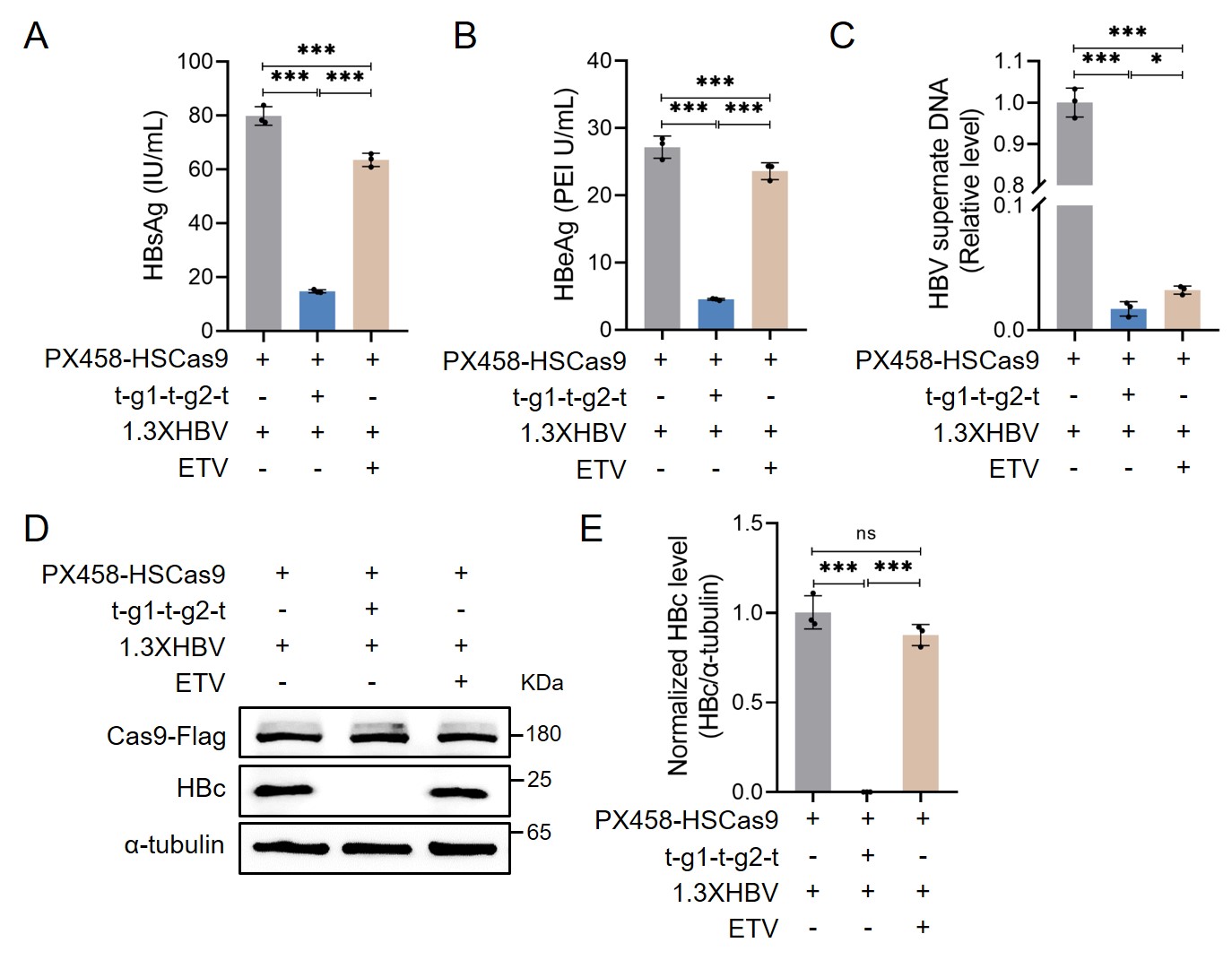
**

**Figure S11.** Compare the ability of HSCas9 and entecavir (ETV) to inhibit HBV replication. The pBB4.5-1.3×HBV, PX458-HSCas9 plasmid, and pU6-t-g1-t-g2-t plasmid or vector control were co-transfected into HepG2 cells, and subsequently treated with ETV (1 µM) or solvent control for 96 h after 6 h of transfection. The levels of (**A**) HBsAg and (**B**) HBeAg in the cell culture supernatants were detected by chemiluminescence immunoassay. (**C**) The level of HBV DNA in the cell culture supernatants was detected by qPCR. (**D**) The levels of Cas9 and HBc proteins were detected by Western blotting, and α-tubulin was used as the internal control. (**E**) The relative level of HBc protein was quantified by ImageJ software. Data are presented as mean ± SD of three independent experiments. **P*<0.05, ****P*<0.001, ns=no statistical significance, two-tailed Student’s *t* test.
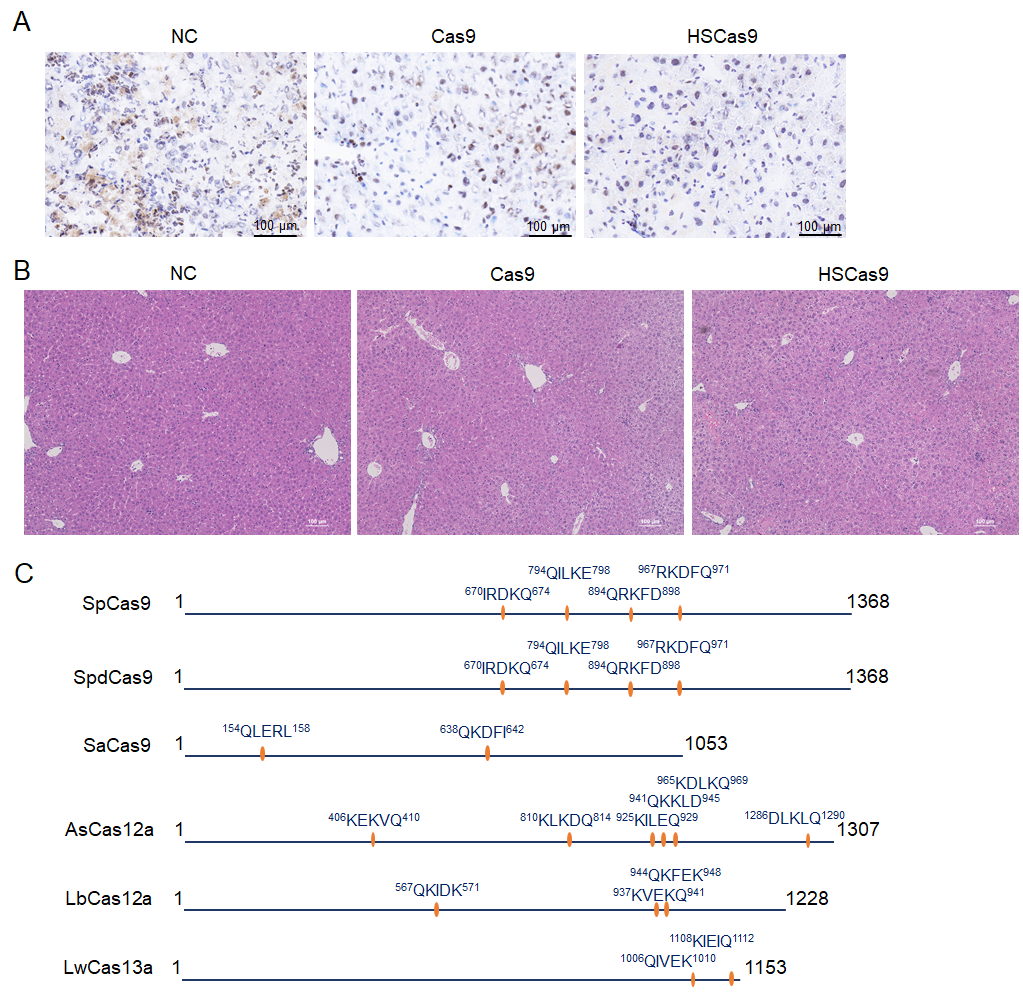


**Figure S12.** The ability of HSCas9 in promoting the CRISPR/Cas9 system to inhibit HBV replication in mice and the distribution of KFERQ-like motif in different Cas proteins. **(A)** At 3 days after hydrodynamic injection, the levels of HBc protein in the liver tissues were detected by immunohistochemical staining. **(B)** At 7 days post of hydrodynamic injection, the effects of CRISPR/Cas9 and CRISPR/HSCas9 systems on liver injury and inflammation of the euthanized mice in each group were detected by hematoxylin-eosin (HE) staining. **(C)** The distribution of KFERQ-like motif in spCas9, spdCas9, saCas9, AsCas12a, LbCas12a, and LwCas13a proteins.

**Supplementary tables**

**Table S1.** The sequences of primers used for plasmid construction.

| Name | Sequences（5′-3′） |
| --- | --- |
| pCDH-HSC70-HA | F:GCTCTAGAGCCACCATGGATGTCCAAGGGACCTGCAGT |
|  | R:CGGGATCCTTAAGCGTAATCTGGAACATCGTATGGGTAATCAACCTCTTCAATGGTGGGCC |
| Cas9-KFERQ-M1 | F:TCAACGGCATCCGGGACGCGGCGTCCGGCAAGACAATCC |
|  | R:GGATTGTCTTGCCGGACGCCGCGTCCCGGATGCCGTTGA |
| Cas9-KFERQ-M2 | F:CATCAAAGAGCTGGGCAGCGCGGCCCTGAAAGAACACCCCGTG |
|  | R:CACGGGGTGTTCTTTCAGGGCCGCGCTGCCCAGCTCTTTGATG |
| Cas9-KFERQ-M3 | F:GCTGAACGCCAAGCTGATTACCGCGGCAAAGTTCGACAATCTGACCAAG |
|  | R:CTTGGTCAGATTGTCGAACTTTGCCGCGGTAATCAGCTTGGCGTTCAGC |
| Cas9-KFERQ-M4 | F:GGTGTCCGATTTCCGGAAGGATGCCGCGTTTTACAAAGTGCGCGAGATC |
|  | R:GATCTCGCGCACTTTGTAAAACGCGGCATCCTTCCGGAAATCGGACACC |

F: forward, R: reverse.

**Table S2.** The sequences of primers used for PCR.

| Name | Sequences（5′-3′） |
| --- | --- |
| *gHBV1* | F: CAAGCCTCCAAGCTGTGCCT |
|  | R: CGGCATAAGGCAGGAAGTTATC |
| *GAPDH* | F: GACAAGCTTCCCGTTCTCAG |
|  | R: GAGTCAACGGATTTGGTCGT |
| *Cas9* | F: GCAGCTGCCTGAGAAGTACA |
|  | R: TGTTCAGCTTCACGAGCAGT |
| *LAMP2A* | F: GAAAATGCCACTTGCCTTTATGC |
|  | R: AGGAAAAGCCAGGTCCGAAC |
| *ACTB* | F: CTACAGCTTCACCACCACGG |
|  | R: TCAGGCAGCTCGTAGCTCTTC |

F: forward, R: reverse.

**Table S3.** The information of antibodies.

| Name | Brand | Product Code | Usage |
| --- | --- | --- | --- |
| Anti-DDDDK-tag | MBL International | M185-3 | WB/Co-IP/IF |
| Anti-GAPDH | Cell Signaling Technology | 2118 | WB |
| α-tubulin | Cell Signaling Technology | 2144 | WB |
| Anti-HA tag | Abcam | ab9110 | WB |
| Anti-HBc | MBL International | T2221 | WB |
| Anti-GFP | MBL International | M048-3 | WB |
| β-actin | Cell Signaling Technology | 4967 | WB |
| Anti-Hsc70 | Abcam | ab51052 | WB/Co-IP/IF |
| Anti-SQSTM1 / p62 | Abcam | ab109012 | WB |
| Anti-LAMP2A | Abcam | ab125068 | IF |
| Anti-Rabbit IgG H&L (Alexa Fluor 647) | Abcam | ab150079 | IF |
| Anti-Mouse IgG H&L (Alexa Fluor 488) | Abcam | ab150113 | IF |

WB: Western blotting, Co-IP: Co-immunoprecipitation, IF: Immunofluorescence.

**Table S4.** The top three predicted off-target sites of gHBV1 in human genome.

| Name | Off-target sites (5’-3’) | Chromosome | Position | Direction | Mismatches |
| --- | --- | --- | --- | --- | --- |
| Site 1 | AGTGTTTGCTGAGGCTTCCGGG | chr8 | 7448818 | - | 3 |
| Site 2 | AGTGTTTCCTGCCGCAAGCTGG | chr20 | 62919057 | - | 3 |
| Site 3 | GGTGTTTGCTGATGCCACCCGG | chr1 | 154346373 | + | 3 |

**Table S5.** The sequences of primers used for amplifying the predicted off-target sites.

| Name | Sequences（5′-3′） |
| --- | --- |
| Off-target site1 | F: CAGTTTTGGGGGCTCTCACTT |
|  | R: AGCAGGGCACTCGCGTGG |
| Off-target site2 | F: AGGGTCTCCTGGCTCATCAAT |
|  | R: TCAGCCTCCCGAAGTGCAG |
| Off-target site3 | F: CTTTGGAAAGGAGGAGGCA |
|  | R: GCAGAAAGTAGGCATAGTCAG |

F: forward, R: reverse.

**Table S6.** The off-target effects of Cas9 and HSCas9 in human genome.

|  |  | Total (reads) | Indels (reads) | Off-target efficiency^a^ | *P* |
| --- | --- | --- | --- | --- | --- |
| Off-target site1 | Cas9 | 89323 | 7 | 0.0078% | 0.248 |
|  | HSCas9 | 97243 | 13 | 0.0134% |  |
| Off-target site2 | Cas9 | 109313 | 8 | 0.0073% | 0.118 |
|  | HSCas9 | 112898 | 3 | 0.0027% |  |
| Off-target site3 | Cas9 | 100138 | 8 | 0.0080% | 0.457 |
|  | HSCas9 | 116649 | 13 | 0.0111% |  |

^a^The off-target efficiency was the number of reads containing indels divided by the total number of reads.

**Table S7.** Whole-genome sequencing analysis of off-targets in hydrodynamic injection mice.

| Mouse | | gRNA | Variant/No. of NGG sites | Variant/No. of NAG sites |
| --- | --- | --- | --- | --- |
| NC | 1# | gHBV1 | 0/206 | 0/326 |
|  |  | gHBV2 | 0/257 | 0/168 |
|  | 2# | gHBV1 | 0/206 | 0/326 |
|  |  | gHBV2 | 0/257 | 0/168 |
|  | 3# | gHBV1 | 0/206 | 0/326 |
|  |  | gHBV2 | 0/257 | 0/168 |
| Cas9 | 1# | gHBV1 | 0/206 | 0/326 |
|  |  | gHBV2 | 0/257 | 0/168 |
|  | 2# | gHBV1 | 0/206 | 0/326 |
|  |  | gHBV2 | 0/257 | 0/168 |
|  | 3# | gHBV1 | 0/206 | 0/326 |
|  |  | gHBV2 | 0/257 | 0/168 |
| HSCas9 | 1# | gHBV1 | 0/206 | 0/326 |
|  |  | gHBV2 | 0/257 | 0/168 |
|  | 2# | gHBV1 | 0/206 | 0/326 |
|  |  | gHBV2 | 0/257 | 0/168 |
|  | 3# | gHBV1 | 0/206 | 0/326 |
|  |  | gHBV2 | 0/257 | 0/168 |
